# High‐Throughput Fluorescence Screening Enables Globally Consistent Identification of ABA Signaling Modulators

**DOI:** 10.1002/advs.202417212

**Published:** 2025-05-08

**Authors:** Yang‐Yang Gao, Chang‐Xin Yang, Hong Wu, Jian‐Hong Li, Ting Wen, Wei Wang, Zhi‐Zheng Wang, Hui‐Min Chen, Rong‐Jie Pei, Zhi‐You Huang, Yu‐Guo Zheng, Guang‐Fu Yang, Xiang‐Yang Li, Ge‐Fei Hao

**Affiliations:** ^1^ State Key Laboratory of Green Pesticide Key Laboratory of Green Pesticide and Agricultural Bioengineering Ministry of Education Center for Research and Development of Fine Chemicals Guizhou University Guiyang 550025 P. R. China; ^2^ State Key Laboratory of Biocatalysis and Enzyme Engineering School of Life Sciences Hubei University Wuhan Hubei 430072 P. R. China; ^3^ State Key Laboratory of Green Pesticide Central China Normal University Wuhan 430079 P. R. China; ^4^ Key Laboratory of Chemical Synthesis and Environmental Pollution Control Remediation Technology Minzu Normal University of Xingyi Xingyi Guizhou 562400 P. R. China

**Keywords:** ABA signaling modulators, fluorescent chemosensor, GhitFluors platform, global high‐throughput screening, lebactin

## Abstract

Frequent exposure to environmental stresses can potentially reduce plant yield by up to 60%. The regulation of abscisic acid (ABA) signaling, especially its core receptors, is crucial for the adaptation of plants to environmental stress. However, efficient identification of ABA‐signaling modulators remains a challenge. Here, a global high‐throughput fluorescence screening (GhitFluors) platform is established to efficiently identify ABA signaling modulators. The GhitFluors platform yields a similar tendency for binding affinity to that determined by classical methods. Diopyridin is effectively identified, a regulator with strong binding affinity to Pyrabactin Resistance 1 (PYR1), and more than 80% inhibitory activity against HAB1, which is equivalent to ABA. Additionally, it can also effectively mitigate the adverse effects of drought on plants. The findings promise the start of a new era for achieving high‐efficiency identification ABA signaling modulators.

## Graphical abstract

This paper establishes a global high‐throughput fluorescence screening (GhitFluors) platform to efficiently identify ABA signaling modulators. It effectively identifies diopyridin, a regulator with strong binding affinity to PYR1, and more than 80% inhibitory activity against HAB1, which is equivalent to ABA. Meanwhile, diopyridin can also effectively mitigate the adverse effects of drought on plants.

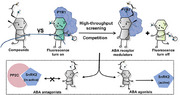

## 1 Introduction

Plants are frequently suffered from the changing environments that affect their growth and productivity.^[^ ^1^ ^]^ Abiotic stresses, such as salinity, heat, flooding, drought, and cold, can significantly reduce crop yields by up to 70%.^[^ ^2^ ^]^ Biotic stresses often cause greater yield losses than other combined factors, potentially leading to substantial economic losses.^[^ ^3^ ^]^ Moreover, plants often face challenges from a combination of abiotic and biotic stresses, leading to a 51–82% reduction in nutrient use, which affects fruit quality.^[^ ^4^ ^]^ Therefore, improving plant resistance to environmental stresses is a major challenge amid efforts to ensure high crop yields and food security.

Abscisic acid (ABA) signaling serves as a crucial messenger for modulating plant adaptation to environmental stresses.^[^ ^5^ ^]^ Specifically, ABA is recognized by 14 receptors, members of the Pyrabactin Resistance 1/PYR1‐Like/Regulatory Components of ABA receptors (PYLs) family in *Arabidopsis thaliana*.^[^ ^6^ ^]^ Upon ABA binding, PYLs interact with group A type 2C protein phosphatases (PP2Cs), blocking their interactions with downstream proteins.^[^ ^7^ ^]^ PP2Cs suppression facilitates the stimulation of SNF1‐related kinase 2s (SnRK2s) and downstream stress responses.^[^ ^8^ ^]^ Further, PYLs activation can improve plant resistance to high temperature, salinity, and drought, as well as manage seed development, flowering, and grain size.^[^ ^9^ ^]^ Additionally, exogenous spraying with modulators can reportedly activate ABA signaling and effectively protect plants from drought.^[^ ^10^ ^]^ Overall, ABA signaling, particularly its receptors, serves as a critical elements for improving plant tolerance to stress.^[^ ^11^ ^]^ Therefore, identification of ABA signaling modulators is a simple and convenient strategy for mitigating plant stress damage.

Recently, a series of direct and indirect techniques have been used to identify ABA‐signaling modulators. The direct technique includes the binding affinity of PYLs to modulators, and the indirect techniques primarily include PP2Cs activity, PYLs‐PP2Cs interaction, and ABA‐responsive gene expression. The binding affinity between the candidates and PYLs, as measured using common technologies, determines whether cyanabactin and opabactin can function as ABA signaling modulators.^[^ ^10^, ^12^ ^]^ Analysis of the effects of AMF4, quinabactin, AA1 and pyrabactin on PP2Cs activity can verify whether their signaling networks align with ABA.^[^ ^12^, ^13^ ^]^ A case in point, the interplay between ABA receptors and PP2Cs has firmly substantiated the identification of AM1 as an ABA receptor agonist.^[^ ^14^ ^]^ The spatio‐temporal gene expression patterns in ABA signaling after treatment with AM1 and AA1 can reportedly be used to assess their interaction proteins or downstream effectors.^[^ ^13^, ^14^ ^]^ In addition, some biosensors and “always‐on” types of chemosensors have been designed for the analysis of ABA distribution within plant tissues and its biological roles; however, they are insufficient for screening ABA‐signaling modulators.^[^ ^15^ ^]^ Overall, the direct and indirect approaches have been widely used to identify ABA agonists or antagonists. However, these technologies are low‐throughput or one‐sidedness, potentially reducing the screening efficiency.

In this study, we aimed to develop a global high‐throughput fluorescent screening (GhitFluors) platform for efficient identification of modulators of ABA signaling. Lebactin was selected as a suitable chemosensor due to its low fluorescent background (19.81), high stability (more than 3 h), large shift (≈80 nm), and appropriate binding affinity to PYR1 (*K*_d_ = 64.9 µm). After interaction with PYR1, the fluorescence intensity of lebactin increased 14.18‐fold, which was attributed to an enhanced intramolecular charge transfer (ICT) mechanism. The *K*_d_ values of PYR1 and known ABA‐signaling modulators, including AMF4, AM1, and pyrabactin, obtained by fluorescent competition and bio‐layer interferometry (BLI), showed a similar tendency, suggesting that the GhitFluors platform can accurately validate the chemical modulators of ABA signaling. After high‐throughput screening of an in‐house library with several hundred amide compounds, a promising modulator diopyridin was quickly identified. The binding affinity of diopyridin with PYR1 (*K*_d_ = 9.5 µm) and its inhibition activity against HAB1 (more than 80% at 50 µm) were equivalent to those of ABA. Treatments with diopyridin could effectively protect kidney beans from drought stress. These findings provide a valuable tool for highly efficient identification of modulators of ABA signaling.

## 2 Results

### 2.1 Traditional Screening for ABA Receptor Modulators is Time‐Consuming and One‐Sidedness

**Figure 1:**
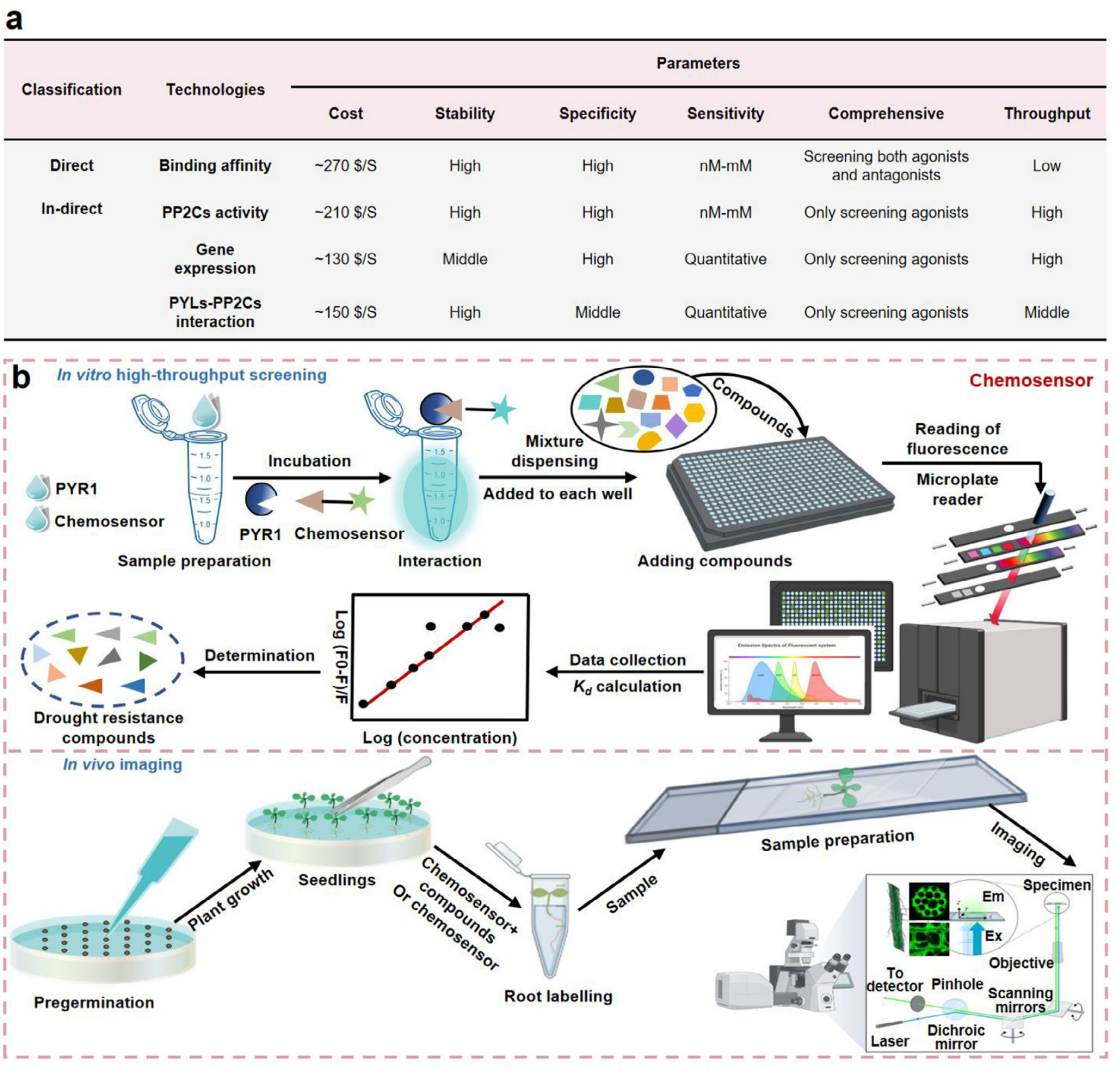
Comparison between conventional techniques for the identification of ABA receptor modulators and the workflow of the high‐throughput fluorescence screening platform. a) Comparison of the advantages and disadvantages of direct (binding affinity of PYLs and compounds) and indirect (PP2Cs activity, ABA‐responsive gene expression, and PYLs‐PP2Cs interaction) traditional techniques for identifying ABA receptor modulators based on cost, stability, specificity, sensitivity, comprehensive and throughput. All techniques showed some common disadvantages including one‐sidedness and low‐throughput. b) The workflow of fluorescent‐based global high‐throughput screening platform for identifying ABA receptor modulators. A chemosensor with suitable fluorescent characteristics is an important requisite for establishing this platform.

To illustrate the need for techniques to identify ABA receptor modulators, we simultaneously compared the workflow and screening parameters between current techniques. Traditional techniques are primarily divided into direct (binding affinity of compounds to PYLs) and in‐direct (PP2Cs activity, ABA‐responsive genes expression, and PYLs‐PP2Cs interaction) ones, according to the type of ABA receptor modulators. As shown in Figure S1 (Supporting Information), measurement of the binding affinity to PYLs necessitates protein expression, purification, an optimal reaction system, and binding affinity analysis.^[^ ^10^, ^12^ ^]^ Evaluating the effect of a compound on PP2Cs activity requires protein purification and enzyme activity detection.^[^ ^12^, ^13^ ^‐d]^ Analysis of ABA responsive‐gene expression involves ribonucleic acid (RNA) extraction, reverse transcription, data measurement, and analysis.^[^ ^13^, ^14^ ^]^ PYLs‐PP2Cs interactions were determined by testing yeast colony growth on SCM/‐4 plates.^[^ ^14^ ^]^ **Figure** **1**a shows the advantages and disadvantages of direct and in‐direct techniques upon evaluating cost, stability, specificity, sensitivity, comprehensive, and throughput. These techniques have some common advantages, including robust stability, specificity, and sensitivity, as well as an acceptable cost of $100–200 per sample.^[^ ^16^ ^]^ For the analysis of comprehensiveness, measuring the binding affinity of compounds with PYLs can achieve the screening of agonists and antagonists simultaneously, whereas other techniques only screen agonists. Meanwhile, the throughput for binding affinity of compounds to PYLs, and the PYLs‐PP2Cs interaction in screening ABA receptor‐modulators remains unresolved. Therefore, it is essential to develop a high‐throughput and global platform to improve the efficiency of any effort aimed at the identification of ABA receptor modulators.

Recognizing the common challenges of traditional technologies, we aimed to establish a global high‐throughput screening platform. Fluorescent chemosensor is a good choice for high‐throughput screening of protein modulators, yielding ≈60% of drugs.^[^ ^17^ ^]^ For example, fluorescent chemosensors targeting the ryanodine receptor,^[^ ^18^ ^]^ chitin synthase,^[^ ^19^ ^]^ type III secretion system,^[^ ^20^ ^]^ GABA,^[^ ^21^ ^]^ and acetohydroxyacid synthase^[^ ^22^ ^]^ have been developed to facilitate the identification of potential hit compounds. In principle, chemosensors (MW < 10 kDa) free rotate swiftly and emit fluorescence, whereas their emission may be changed upon binding to proteins. As shown in Figure 1b, designing fluorescent chemosensors can bind with the ABA receptor and co‐incubate in a centrifuge tube. Then, changes in emission wavelength and fluorescence intensity can be observed. The uniform mixture was evenly distributed into a 384‐well plate, a chemical library was incorporated, and the fluorescence parameters were measured using a microplate reader. Compounds demonstrating a higher binding affinity for PYR1 than chemosensors can replace the latter in the binding pocket, leading to changes in fluorescence characteristics. Meanwhile, the binding affinities (*K*_d_) of the selected compounds were calculated, which is crucial for identifying ABA receptor modulators. Additionally, the identification of ABA receptor modulators was confirmed by in vivo testing. Ten‐day‐old seedlings were submerged in a mixture of compounds and chemosensors. Fluorescence intensity changes in roots can be observed under detection devices, such as fluorescent microscope, which provide strong evidence for screening ABA receptor modulators in vivo. Above‐all, a suitable fluorescent chemosensor is critical for this platform to obtain global high‐throughput screening of ABA receptor modulators.

### 2.2 A Global High‐Throughput Fluorescence Screening (GhitFluors) Platform

**Figure 2:**
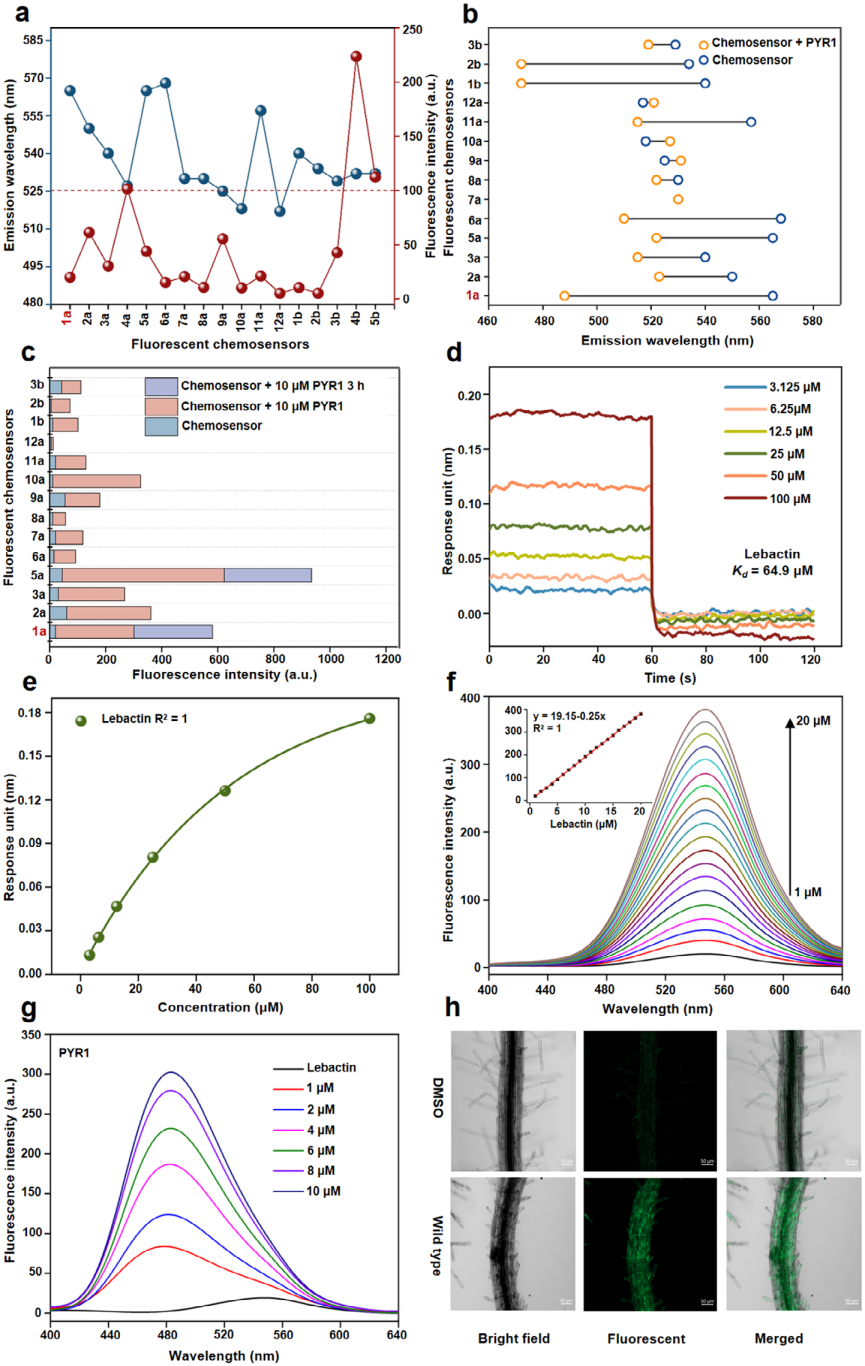
Identification of the suitable fluorescent chemosensor and mixture ratio of lebactin‐PYR1 for the GhitFluors platform. a) Emission wavelength and fluorescence intensity of 17 tested chemsensors. Chemosensors with fluorescence intensity lower than 100 were selected. b) Fluorescence shift of chemosensors after interaction with PYR1. Suitable chemosensors should exhibit an emission wavelength shift exceeding 30. c) Fluorescence stability of chemosensors after co‐incubation with PYR1 for 3 h. Among all tested chemosensors, compound 1a (lebactin) exhibited the highest stability. d) The *K*_d_ values of lebactin binding with PYR1 measured by BLI. Lebactin showed moderate binding affinity to PYR1 with a *K*_d_ value of 64.9 µm. e) The correlation between lebactin concentrations and the corresponding response unit. The high R^2^ value implied the rationality of lebactin concentrations and precise of *K*_d_ value. f) Fluorescence intensity and emission wavelength obtained from 1 to 20 µm of lebactin. The 1 µm concentration of lebactin was selected as the suitable. g) Changes in fluorescence intensity and emission wavelength of the mixture system for lebactin‐PYR1 (1–10 µm). The 8 µm concentration of PYR1 was selected as the most favorable. h) In the in vivo labeling assay, roots exhibited bright green fluorescence after treated with lebactin, which demonstrated that lebactin is capable of interacting with PYR1 in complex biological environments.

Based on demands of the GhitFluors platform, a suitable chemosensor was selected. It should have a low fluorescence background, high fluorescence shift and stability, and appropriate binding affinity, as these determine the accuracy, feasibility and, the sensitivity of this platform, respectively. The structures of the 17 tested chemosensors (compounds 1a–12a, 1b–5b) obtained from our fluorescent compound library were shown in Figures S2 and S3 (Supporting Information).^[^ ^15d^ ^]^ All chemosensors exhibited a strong absorption peak at 310–370 nm within the UV–vis spectra in PBS buffer solution (Table S1, Supporting Information). First, we evaluated the fluorescence background of all chemosensors by using 310–370 nm as excitation wavelength. In **Figure** **2**a, these chemosensors exhibited yellow‐green emission spectra spanning 512–575 nm. To reduce background interference, chemosensors (compounds 4a, 4b, and 5b) with fluorescence intensities above 100 were eliminated, while the remaining chemosensors had an acceptable fluorescence background lower than 61.2. Subsequently, the emission wavelengths of the chemosensors were measured after binding to PYR1, as shown in Figure 2b. Compounds 2a, 3a, 7a, 8a, 9a, 10a, 12a, and 3b showed fluorescence shifts lower than 27, implying possible mutual fluorescence interference between chemosensor‐free and chemosensor‐PYR1 complexes that decreased screening accuracy. Only compounds 1a, 5a, 6a, 1b, and 2b, with a blueshift of more than 50 nm, were retained. Meanwhile, the fluorescence stability of chemosensor‐PYR1 mixture system, as displayed in Figure 2c. Compound 1a (lebactin) was the most stability chemosensor, with slight changes in fluorescence intensity (from 280.81 to 280.32), while the other chemosensors showed no or significantly decreased fluorescence at all. In addition, the binding affinity of compound 1a (lebactin) for PYR1 was evaluated by bio‐layer interferometry (BLI). It was observed that lebactin can bind to PYR1 with a moderate *K*_d_ value, suggesting that compounds with *K*_d_ value lower than 64.9 µm can replace lebactin in the binding pocket of PYR1 (Figure 2d). The saturation curve for lebactin concentration and the corresponding response units illustrated the reliability of *K*_d_ value (Figure 2e). All results indicated that lebactin was the most suitable fluorescent chemosensor for the GhitFluors platform, owing to its low fluorescence background, high fluorescence shift, favorable stability and appropriate binding affinity for PYR1.

To ensure the accuracy of the GhitFluors platform for measuring binding affinity, the appropriate mixing ratio of lebactin and PYR1 was determined.^[^ ^23^ ^]^ The most suitable concentration of lebactin and the titration efficiency of PYR1 were determined based on the correlation between fluorescence intensity and binding affinity. First, the optimal concentration of lebactin was determined by measuring its fluorescence intensity from 1 to 20 µm. Figure 2f presented the fluorescence intensity enhanced with increasing lebactin concentration. A robust linear correlation was observed between fluorescence intensity and lebactin concentrations. To reduce the fluorescence interference of lebactin, 1 µm was selected as the suitable concentration, as it showed the lowest fluorescent intensity of 20.27. Simultaneously, the titration efficiency of PYR1 was assessed using the concentration‐dependent titration curve, as shown in Figure 2g. Fluorescence intensity of the lebactin‐PYR1 mixture initially increased rapidly, but decreased with increasing PYR1 concentrations. No significant difference in fluorescence intensity between PYR1 at 10 and 8 µm, implying that 8 µm represents the optimal concentration. In addition, in vivo fluorescent labeling assays were performed. A strikingly intense green‐fluorescence emission from lebactin‐treated *Arabidopsis* roots was observed under a confocal microscope, indicating that lebactin has great potential for screening ABA receptor modulators under complex biological conditions (Figure 2h). These data substantiated that lebactin at 1 µm and PYR1 at 8 µm are adequate concentrations for constructing this platform.

### 2.3 GhitFluors Shows High Consistent with Classical Methods Used for Determining Binding Affinity

**Figure 3:**
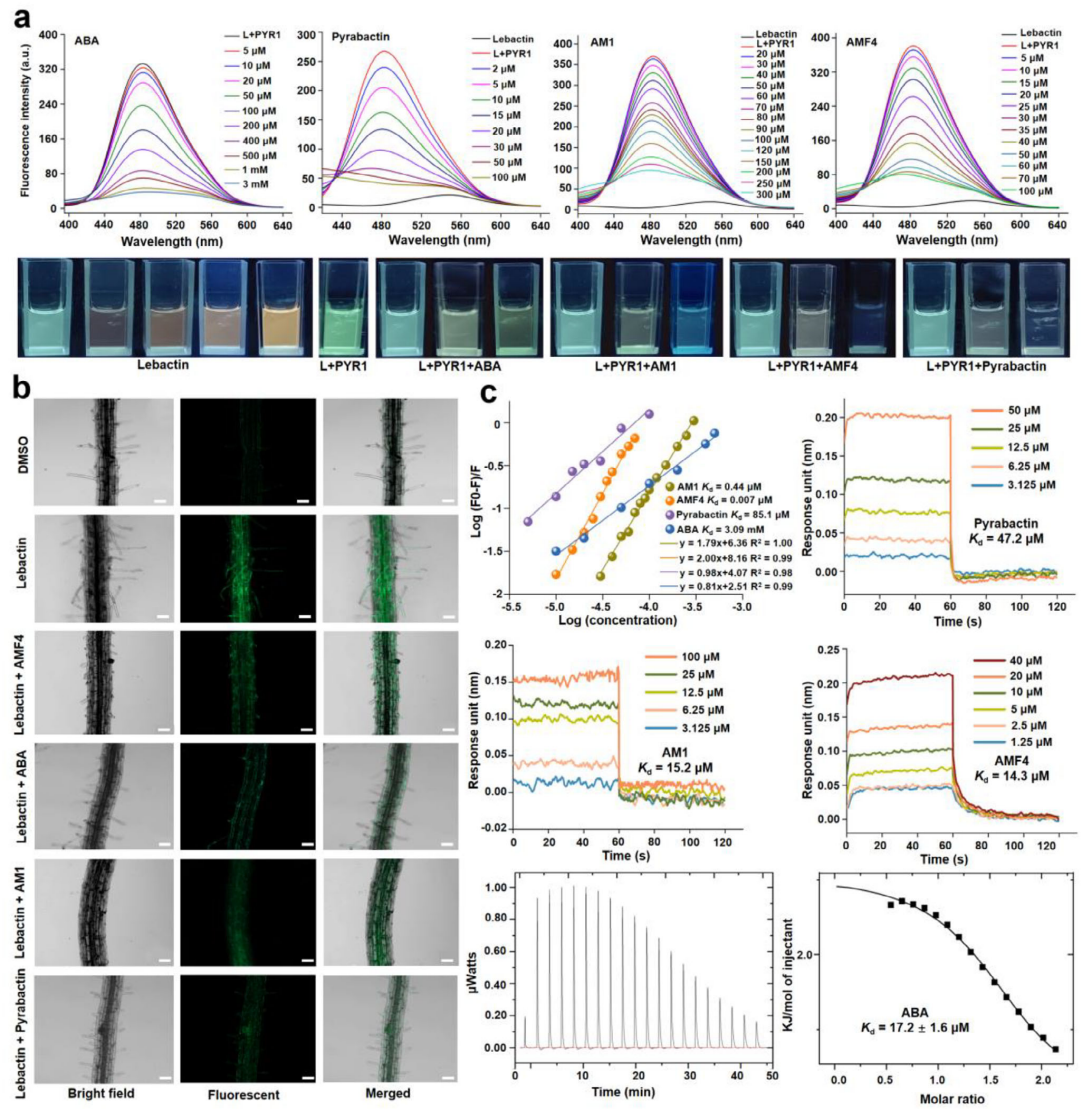
The GhitFluors platform can precisely validate the known ABA receptor modulators including ABA, pyrabactin, AM1 and AMF4. a) The in vitro fluorescence competition assay of ABA, pyrabactin, AM1 and AMF4 with lebactin. The significant changes in fluorescence intensity and color demonstrated that the known ABA mimics prevented the binding of lebactin to PYR1. b) Confocal imaging of 7‐day old *Arabidopsis* roots treated with lebactin, and the mixture of lebactin with ABA, pyrabactin, AM1 and AMF4. The significantly decreased green fluorescence implied that lebactin screened ABA receptor modulators in vivo. Bars: 50 µm. c) The *K*_d_ values of ABA, pyrabactin, AM1 and AMF4 for PYR1 were obtained by fluorescence competition and BLI/ITC assays. The similar tendency between *K*_d_ values measured by the two methods indicated that the GhitFluors platform effectively identified the known ABA receptor modulators.

To evaluate the feasibility of GhitFluors platform for screening ABA receptor modulators in vitro, we first conducted fluorescence competition assay using known compounds. As shown in **Figure** **3**a, the competition efficiencies of ABA, pyrabactin, AM1, and AMF4 were detected by recording the changes in fluorescence intensity and emission wavelength. As the concentrations of the four tested compounds increased, fluorescence brightness and emission wavelength of the mixed systems first changed obviously and then slowly. Upon reaching 3 mm, 100, 300, and 100 µm of ABA, pyrabactin, AM1, and AMF4, respectively, fluorescence intensity was close to the threshold values, implying that the concentrations have reached the saturation level. Photographs obtained from fluorescence competition assays showed that green fluorescence intensity increased with the elevated concentrations of lebactin. After co‐incubation with PYR1 for 10 min, a significant enhancement in green fluorescence intensity was observed. The addition of ABA, pyrabactin, AM1, and AMF4 into the lebactin‐PYR1 mixture system resulted in a significant alteration in fluorescence intensity and color, consistently with the fluorescence titration curves. These results demonstrated that the GhitFluors platform can screen ABA receptor‐modulators in vitro.

Fluorescence competition assays were also performed in *Arabidopsis* to analyze the potential of the GhitFluors platform for screening ABA receptor modulators in vivo. As displayed in Figure 3b, no obvious fluorescence signal was detected in DMSO‐treated plants. An obvious green fluorescence emission was observed in lebactin‐treated wild‐type *Arabidopsis*, while only a weak green‐fluorescence signal was detected in *Arabidopsis pyr1* mutants (Figure S5, Supporting Information). This phenomenon may be attributed to the formation of lebactin‐PYR1 complex. After co‐incubation for 72 h with ABA, pyrabactin, AM1, and AMF4, a significant decrease green fluorescence signal in lebactin‐treated *Arabidopsis* was detected (*p* = 0.0001) (Figure S6, Supporting Information), indicating that these compounds can competitively prevent lebactin binding to PYR1 in a biological environment. These findings suggested that the GhitFluors platform can screen ABA‐receptor modulators under complex biological conditions.

To determine the consistency of GhitFluors platform with traditional technologies for quantitatively identifying known ABA receptor modulators, we compared *K*_d_ values obtained using these methods. As illustrated in Figure 3c, the *K*_d_ values of fluorescence competition assays were calculated as described by Xing et al. (2018).^[^ ^24^ ^]^ The log (concentration) of four known ABA receptor modulators and their corresponding log (F_0_‐F)/F showed a strong correlation with R^2^ values more than 0.97. After linear regression and Scatchard equations analysis, the *K*_d_ values of ABA, pyrabactin, AM1 and AMF4 were 3.09 mm, 85.1, 0.44, and 0.007 µm, respectively. Second, the binding affinities of the four known ABA receptor modulators to PYR1 were evaluated using BLI or Isothermal titration calorimetry (ITC). Among them, the binding affinities of pyrabactin, AM1 and AMF4 to PYR1 were determined by BLI owing to their molecular weight over than 300, which showed *K*_d_ values of 47.2, 15.2, and 14.3 µm, respectively. The highly significant correlation (≈0.99) between compound concentration and the corresponding responses implied the accuracy of *K*_d_ measurement (Figure S7, Supporting Information). The binding affinity of ABA to PYR1 could not be determined by BLI because a molecular weight below 300 failed to induce a wavelength shift in the SSA biosensor upon binding with PYR1. After ITC measurements, ABA showed a *K*_d_ value of 17.2 ± 1.6 µm. The *K*_d_ values obtained from GhitFluors platform followed a tendency similar to that evaluated by BLI, showing the following pattern: pyrabactin > AM1 > AMF4. In addition, we did not perform regression analysis on *K*_d_ values to determine the consistency between the GhitFluors platform and the BLI or ITC assays owing to the lack of data (only four data points) and different traditional techniques. Compared with the BLI or ITC methods, the advantages of GhitFluors platform in evaluating the binding affinity of ABA mimics with PYR1 include 1) High throughput analysis: the GhitFluors platform could analyze at least 96 different treatments at once, while either BLI or ITC could only analyze one treatment at a time; 2) Low cost: GhitFluors platform is at least 10 times less expensive than either BLI or ITC; 3) Simple operation: Compared with BLI or ITC, the operation of microplate reader is common and simple. Together, these results suggested that the GhitFluors platform is accurate and efficient for validating known ABA‐receptor modulators.

### 2.4 Efficient Discovery of a Promising ABA Mimic to Improve Drought Tolerance

**Figure 4:**
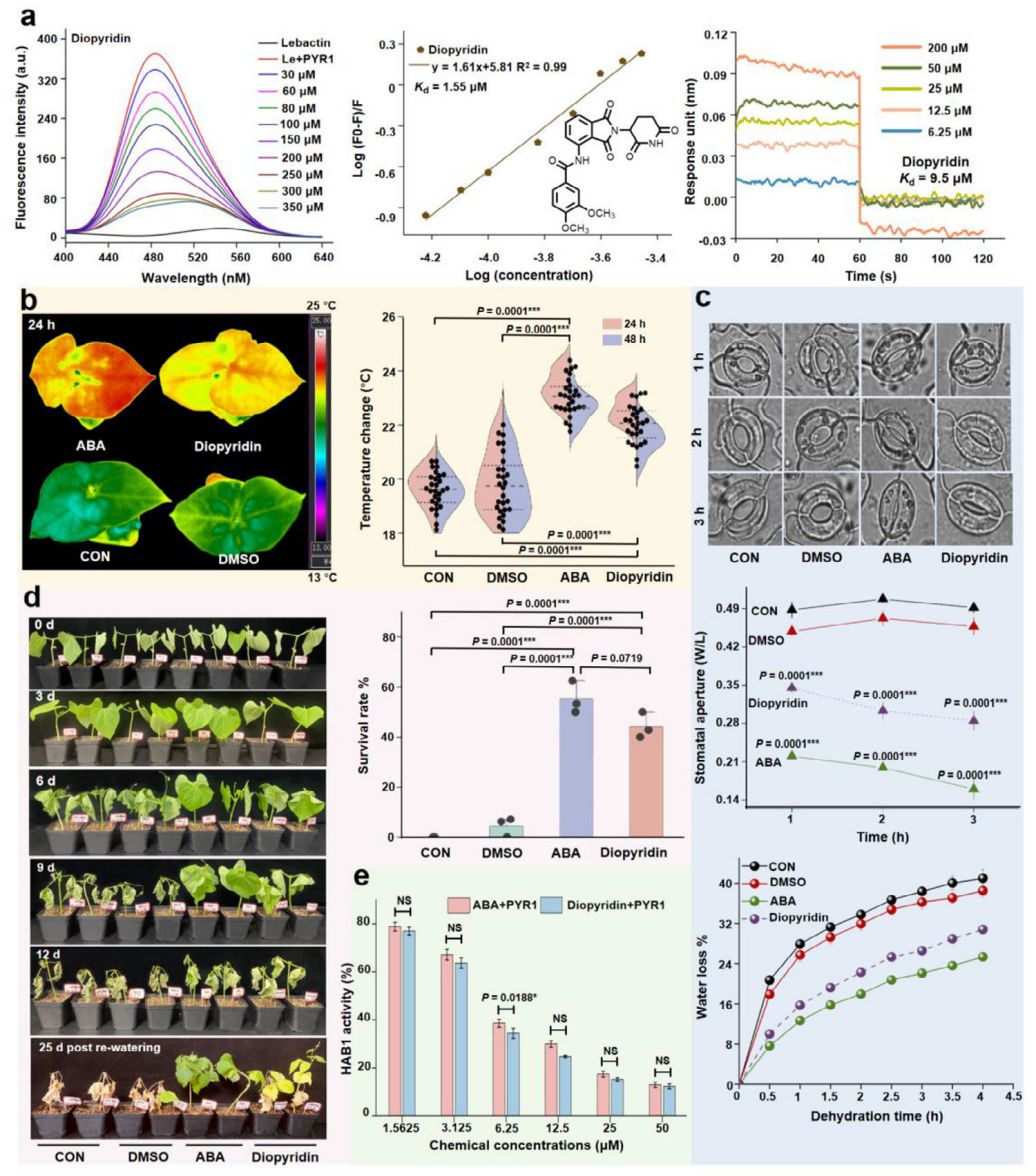
Binding affinity of diopyridin with PYR1 and its effect on stomatal closure, water loss, leaf temperature, and drought resistance. a) Measurement the binding affinity of PYR1 with diopyridin by fluorescence competition and BLI. Diopyridin showed favorable binding affinity with PYR1. b) Plants were captured with an IR camera at 24 and 48 h post‐treatment. Diopyridin could improve leaf surface temperature, implying it has high potential for reducing transpiration rate of plants. c) Stomata were captured using an optimal microscope, and their ratio of width to length were calculated. Water losses after ABA and diopyridin treatments were calculated. The decreased stomatal aperature and water losses were consistent with leaf temperature, indicating diopyridin can improve water retention. d) Evaluating the activity of diopyridin on improving plant drought resistance and the survival rates of kidney beans were calculated 25 days after watering resumption. Diopyridin can effectively protect plant from drought stress. All data are presented as the mean ± SE, *n* = 3. ^*^ *p* < 0.05, ^**^ *p* < 0.01, and ^***^ *p* < 0.001. e) PYR1 mediates the inhibition of HAB1 activity induced by ABA and diopyridin in phosphatase functionality assessments. Diopyridin showed similar inhibitory activity against HAB1 as that of ABA, indicating diopyridin can induce plant drought‐tolerance by stimulating HAB1 activity. Data are presented as the mean ± SE, *n* = 3. ^*^ *p* < 0.05, NS: No significant.

To validate the practicality of GhitFluors platform for global high‐throughput identification of ABA receptor modulators, we used this platform to analyze a library comprising several hundreds‐amide compounds. First, we evaluated *K*_d_ values of several hundreds amide‐compounds using fluorescence competition assays. After analysis of fluorescence intensity, 6 primary hits including 145a, 260a, 361a, 368a, 470a and 173b (diopyridin) were identified as showing favorable binding affinities for PYR1, with *K*_d_ values ranging from 1.55 to 37.75 µm, as shown in Table S2 (Supporting Information). Their structures were displayed in Figures S8–S19 (Supporting Information). Meanwhile, binding affinities of the six compounds to PYR1 were evaluated using ITC or BLI assays to validate the screening results. The *K*_d_ values for these compounds ranged from 7.06 to 293.26 µm (Table S2, Supporting Information). Comparing *K*_d_ values of the six compounds, compound 173b (diopyridin) exhibited the most favorable binding affinity for PYR1 in the fluorescence competition and BLI assays. As depicted in **Figure** **4**a, a significant decrease in fluorescence intensity was observed as the concentration of diopyridin increased, ranging from 30 to 350 µm. When the concentration of diopyridin reached 350 µm, fluorescence intensity was close to the threshold. Regression analysis of log (concentration) for diopyridin and its corresponding log (F_0_‐F)/F showed a strong correlation with an R^2^ value of 0.99. According to linear regression and Scatchard equations, diopyridin exhibited a *K*_d_ value of 1.55 µm. The binding affinity of diopyridin for PYR1 was confirmed using BLI with a *K*_d_ value of 9.50 µm. The favorable correlation between concentrations of diopyridin and their response units (R^2^ = 0.986) suggested a high reliability of *K*_d_ values (Figure S7, Supporting Information). In addition, we also tested the binding affinities of diopyridin with other representative PYLs, including dimer (PYL1, PYL2), and monomer (PYL4, PYL5, and PYL10‐PYL12) that had *K*_d_ values ranging from 0.97 to 32.8 µm, and 1.6 to 243.7 µm, respectively (Figure S20, Supporting Information). These results indicated that diopyridin maybe a promising ABA‐receptor modulator with a substantial binding affinity for PYR1.

To quickly identify whether diopyridin can reduce water transpiration of plants, leaf temperature was first measured after treated with diopyridin.^[^ ^25^ ^]^ As shown in Figure 4b, the leaf temperature of kidney bean plants after being treated with diopyridin was captured and recorded using an IR camera. Before treatment, no obvious differences in leaf temperature were observed in any of the plants. At 24 and 48 h post‐treatment, ABA and diopyridin increased the leaf temperature compared with the control groups. Importantly, the effects of diopyridin can persist for 2 days, implying its chemical stability. Analysis of average leaf temperatures for all treatments collected every hour for 2 days post treatment, ABA and diopyridin can significantly increase leaf temperature by ≈3.2 and 2.2 °C compared to control groups (*p* = 0.0001), respectively. The increase in leaf temperature suggested that diopyridin has high potential for reducing water transpiration of plants.

Two parameters, including stomatal aperture and water losses, were used to evaluate water retention of plants.^[^ ^13^, ^14^ ^]^ First, we examined the effect of diopyridin on stomatal aperture, as displayed in Figure 4c. Stomatal aperture decreased with the increasing in ABA and diopyridin treatment time. At 3 h post ABA and diopyridin treatments, almost all stomata completely closed. By comparing the width:length ratios with those of the control plants, ABA and diopyridin‐treated groups resulted in a significant reduction in stomatal aperture. Second, water losses after diopyridin treatment was consistent with the corresponding response of stomatal aperture. After treated with diopyridin and ABA, water losses of kidney bean leaves decreased gradually with the prolongation of time. Compared with the control groups, ABA and diopyridin could significantly reduce water losses with rate of 7.63–25.35% and 10.01–30.81%, respectively. These results strongly indicated that diopyridin can improve water retention in plants by regulating stomatal aperture.

To validate the activity of diopyridin to improve drought resistance, we performed drought assays on whole kidney beans. As shown in Figure 4d, kidney bean plants treated with ABA and diopyridin displayed enhanced resistance to drought compared with the control groups. Nine days after water withdrawal, the majority of kidney beans for the control groups has wilted, while those treated with ABA and diopyridin remained unwilting and exhibited robust growth. Twelve days after water withdrawal, most of the plants in the control groups have died, whereas most of the ABA or diopyridin‐treated ones appeared wilted. Survival rates for ABA and diopyridin treatments after 25 days post re‐watering were significantly higher than those under control conditions (only 4.5%) with *p* = 0.0001. Meanwhile, the survival rates of diopyridin‐treated plants showed no significant difference with that of ABA‐treated plants (*p* = 0.0719). Compared with ABA and known ABA mimics, diopyridin had some advantages such as 1) diopyridin was greater stability than ABA under UV with degradation rate of 63.7% and 30.9% at 96 h, respectively (Figure S21, Supporting Information); 2) the known ABA mimics have effect on seed germination or root growth, while diopyridin has no effect (Table S3 and Figure S22, Supporting Information). These results demonstrated that diopyridin effectively enhanced plant drought resistance.

To elucidate the mechanism of diopyridin for improving plant drought resistance, we measured its inhibitory activity against HAB1. As shown in Figure 4e, different concentrations of diopyridin were tested in the HAB1 phosphatase inhibition assay and HAB1 phosphatase activity was progressively inhibited (21.88–87.79%) with increasing concentrations of diopyridin. The inhibitory activity of diopyridin against HAB1 was equivalent to that observed in 25 µm ABA‐treated plants (*p* < 0.0188), which showed inhibition rates more than 80%. The enzyme inhibitory activity also indicated that diopyridin had considerable activity along with ABA in inducing HAB1 binding of PYR1.

### 2.5 The Enhanced ICT Effect Maybe the Screening Mechanism of GhitFluors Platform

**Figure 5:**
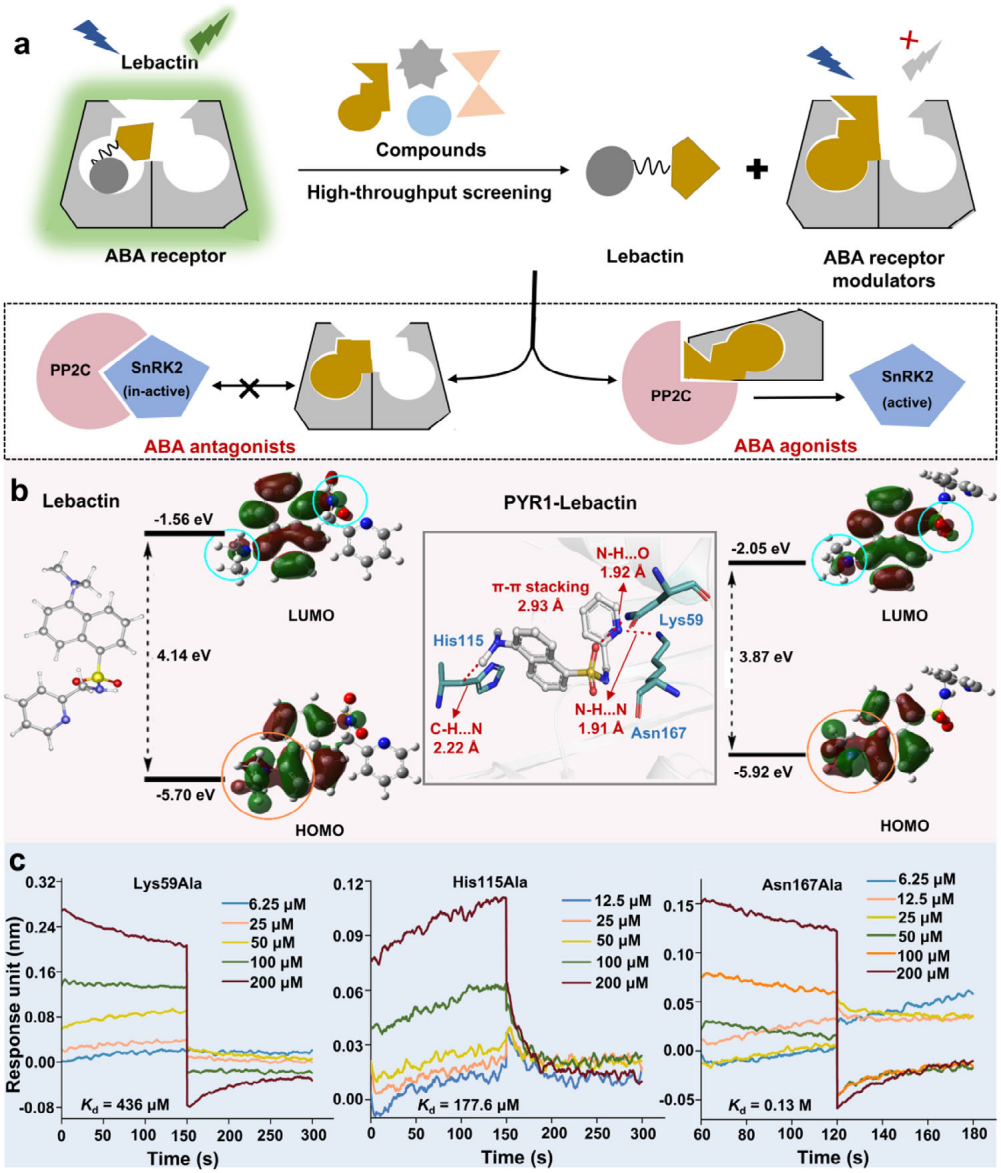
Mechanism and work principle of the GhitFluors platform for identifying ABA receptor modulators. a) The principle of the GhitFluors platform for high‐throughput screening of ABA receptor modulators. Compounds with higher binding affinity with PYR1 can compete with lebactin that resulting in the changes of fluorescence intensity and emission wavelength. b) The phenomenon of geometric relaxation ensuing photoexcitation and photoemission, along with the frontier molecular orbitals (FMOs) involved in the vertical excitation and emission of lebactin‐free and lebactin‐PYR1 complexes. The enhanced ICT between dimethylamino and naphthalene explained the more brightness of lebactin after binding with PYR1. c) The binding affinity of lebactin and PYR1 with mutations of Lys59Ala, His115Ala, and Asn167Ala. The decreased binding affinity of mutant PYR1 indicated that Lys59, His115, and Asn167 are the main amino acid sites for lebactin interaction.

The GhitFluors platform was effective for global high‐throughput screening of ABA receptor modulators with “off‐on” fluorescence switchability. The working principle of this platform involves the analysis of fluorescence intensity and shift after adding candidates, as illustrated in **Figure** **5**a. Clearly, candidates with considerably higher binding affinities for PYLs can replace lebactin to occupy the binding pocket of PYLs, which drastically changes the fluorescence intensity and emission wavelength of the screening system. In other words, candidates possessing a lower binding affinity for PYLs cannot alter the interaction of lebactin and PYLs, as well as the fluorescence parameters do not change. Therefore, GhitFluors platform could be used to simultaneously screen both ABA agonists and antagonists.

To illustrate the screening mechanism of this platform, the quantum mechanics/molecular mechanics (QM/MM) were determined. As shown in Figure 5b, the protonated state of lebactin was deemed necessary to replicate the biological conditions of lebactin and get the most precise ICT model. Through rigorous computations, the energy gap between the lowest unoccupied molecular orbital (LUMO) and the highest occupied molecular orbital (HOMO) was confirmed as the main frontier molecular orbitals (FMOs) involved in the transformation of lebactin‐free and PYR1‐lebactin complexes. The HOMO and LUMO of lebactin‐free was lower than those of the lebactin‐PYR1 complexes. To explain the change in energy gap between lebactin‐free and PYR‐lebactin complex, the binding conformation of lebactin with PYR1 was discussed. Besides hydrophilic interactions contributing to the stability of lebactin within the pocket, there are three main interactions among lebactin and residues that change the distribution of electron clouds in the HOMO and LUMO. Among them, residues Asn167 and Lys59 provided electron‐donating interactions for lebactin, and the residue His115 withdrew electrons in the direction of the dimethylamino group in lebactin via electron‐withdrawing interactions. By comparing the HOMOs of lebactin as a free molecule and after binding with PYR1, the distribution of electron cloud in HOMO of lebactin in the pocket was found to be more concentrated in the orange circle. More concentrated electrons make the spaces of electron cloud in the HOMO and LUMO of lebactin‐PYR1 complex larger than those in the lebactin‐free, due to substantial electron repulsion. There are two electronic effects of PYR1 that reduce the gap of FMOs for lebactin. First, the electron‐withdrawing effect of His115 makes the electron cloud more concentrated in the dimethylamino group and naphthalene ring, and a more concentrated electron cloud can lead to more significant electron repulsion. Second, the electron‐donating effects of Asn167 and Lys59, and *π*–*π* stacking between benzene and naphthalene rings provide larger space for the electron cloud of HOMO in lebactin. These results indicated that the lower energy gap was responsible for the higher brightness of lebactin‐PYR1 complex.

To further clarify whether Lys59, His115, and Asn167 are key amino acid residues for electron transfer between PYR1 and lebactin, we measured the binding affinity of lebactin with the mutant PYR1. We have mutated the three major amino acids into Ala. After measuring, the mutant PYR1 showed significantly decreased binding affinity with lebactin, which showed *K*_d_ values of 436, 177.6 µm and 0.13 m for Lys59Ala, His115Ala, and Asn167Ala mutations, respectively. Among them, Asn167Ala mutation showed the most important role for lebactin‐PYR1 complex with reduction fold more than 1000 times. These results indicated that Lys59, His115, and Asn167 are important amino acid residues for the higher brightness of lebactin after binding with PYR1.

## 3 Discussion

As shown in the workflow of the GhitFluors platform, it could presumably identify any compound that has a higher binding affinity for PYR1 than that of lebactin. However, the selected candidates are ABA receptor antagonists or agonists that remain unknown. To accurately distinguish ABA agonist or antagonist, it is imperative to develop an enzyme‐responsive fluorescent chemosensor that can be used to detect the activity of PP2Cs.^[^ ^26^ ^]^ In addition, this platform showed an “off‐on” fluorescence mode that did not conform to conventional screening practice. Therefore, a fluorescent chemosensor with an improved energy gap after binding with PYR1 can exhibit an “on‐off” mode that is more suitable for further improvement of this platform in the future.

GhitFluors platform can be used to screen ABA‐receptor modulators in vitro and in vivo. There are some differences in the in vitro and in vivo tests: 1) Screening throughput: Only the in vitro test could realize high‐throughput screening; 2) Screening accuracy: The complex biological environments significantly decreased the screening accuracy of in vivo tests; 3) Binding affinity calculation: There are standardized methods for calculating *K*_d_ values in the in vitro test, while there are no certain methods for the calculation in the in vivo tests. Nevertheless, the in vitro test provided guidance for the in vivo test, including compound concentrations, excitation wavelength, and incubation process. Therefore, the in vitro tests can be used to high‐throughput screen ABA‐receptor modulators, while the in vivo test only was used to verify the screening results of the in vitro test.

Different in vivo testing systems could affect the accuracy, sensitivity and specificity of GhitFluors platform, as it can be affected by environmental conditions. For example, plants could produce more ABA under drought or other abiotic stresses, then ABA will occupy the binding pocket of PYR1 that may decrease the screening accuracy. The metabolic capacity and background interference of different plants could affect the sensitivity of this platform. For instance, plants that vary in their ability to metabolize compounds might lead to changes in the concentration of lebactin, which may reduce the fluorescence response of plants. Meanwhile, the spontaneous fluorescence of plant leaves coincides with the emission wavelength of lebactin that may interfere the modulators screening. In addition, the difference of key amino acid residues of binding pocket in PYR1 protein for various plants could affect the specificity of this platform. Therefore, we will further explore the applicability of this platform in different in vivo systems.

An excellent compound for improving plant drought resistance needs to meet the following requirements: high stability, favorable penetration, satisfactory activity, and long lasting. Compared with ABA, diopyridin showed greater stability, considerable persistence, and activity. However, diopyridin only at 100 µm can exhibit similar activity to that of ABA at 40 µm for increasing drought‐tolerance of plants. This phenomenon maybe attributed to poor absorption and translocation of diopyridin. There are several measures for improving the penetration rate of diopyridin, such as adding an appropriate concentration of organosilicone or oil adjuvants to increase its wetting and spreading; making nano‐formulations to realize its precise release and translocation efficiency; modifying it structure by glycosylation and amination. Therefore, future studies should focus on optimizing the penetration characteristics of diopyridin.

## 4 Conclusion

Overall, our work provides an effective GhitFluors platform for the global high‐throughput screening of ABA receptor modulators. Lebactin serves as a suitable chemosensor with high fluorescence stability, low background, larger shift, and favorable binding affinity for PYR1. The enhanced fluorescence intensity of lebactin after binding to PYR1 was attributed to an increase in the ICT mechanism. A similar trend of *K*_d_ values for known ABA receptor modulators to PYR1, as measured by fluorescence competition assays and traditional technologies, suggested that the GhitFluors platform exhibited high screening accuracy. After screening a library comprising several hundreds‐amide compounds using this platform, a promising molecule diopyridin was quickly identified. Its binding affinity to PYR1 (*K*_d_ = 9.5 µm) and inhibition activity against HAB1 were equivalent to those of ABA. Diopyridin is a promising compound for inducing stomatal closure, reducing leaf transpiration and mitigating drought stress of plants. This platform will open up a new avenue for global high‐throughput screening of ABA receptor modulators.

## 5 Experimental Section

### Cloning and Expression of PYR1

The complete PYR1 sequence was amplified using an *Arabidopsis* cDNA template and subsequently cloned into the expression vector pET28a.^[^ ^27^ ^]^ This plasmid was transformed into *Escherichia coli* BL21 (DE3) cells. PYR1 was expressed under 16 °C for 15 h induced by 0.5 m of isopropyl β‐D‐thiogalactopyranside. Nickel column affinity chromatography (Ni‐NTA) was used for protein purification. Approximately 1 mg mL^−1^ of pure PYR1 was obtained and preserved in glycerol at −80 °C. Meanwhile, PYR1 with mutations of Lys59Ala, His115Ala, and Asn167Ala, respectively were also obtained by the same methods.

### Fluorescent Properties of the Designed Chemosensors

A comprehensive spectral profile of ultraviolet absorption for fluorescent chemosensors was established, with the maximum ultraviolet absorbance corresponding to its excitation wavelength. The emission wavelength of each chemosensor was determined by scanning the excitation wavelengths. Chemosensors and PYR1 were thoroughly mixed in a buffer solution (10 mm PBS, pH 7.4), and incubated for 2 min at room temperature. Changes in the emission wavelength and fluorescence intensity were then observed.^[^ ^28^ ^]^ To analyze the fluorescence stability of PYR1‐chemosensors, the mixed solution was incubated at 25 °C for another 3 h, and fluorescence intensity was monitored.

### Validation of the Fluorescent Chemosensor‐Based Screening Platform

The binding affinities to PYR1 of known ABA receptor modulators, including ABA, pyrabactin, AM1, and AMF4 to PYR1 were measured using fluorescence competition assays to evaluate the screening accuracy of the established platform. Before validation, 1 µm lebactin and 8 µm PYR1 were co‐incubated for 2 min, then a series of concentrations of the abovementioned compounds were added. The *K*_d_ values were calculated using the following formula, where *K*, n, and Q represent the binding constant (*K*_a_), number of binding sites, and compound concentrations, respectively. Additionally, the fluorescence characteristics of competition assays were also photographed. All treatments included lebactin at 1, 2, 5, 10, and 15 µm, the mixture of 1 µm lebactin and 8 µm PYR1, lebactin + PYR1 + ABA (20 and 400 µm), lebactin + PYR1 + pyrabactin (20 and 100 µm), lebactin + PYR1 + AM1 (150 and 300 µm), and lebactin + PYR1 + AMF4 (50 and 100 µm).

(1) $$\mathrm{lg}\left(\frac{F_{0}-F}{F}\right)=\mathrm{lg}K+n\mathrm{lg}\left[Q\right]$$

(2) $$K_{d}=1/K_{a}$$

### In‐house Chemical Database Screening Assay

To validate the feasibility of this platform for the high‐throughput screening of ABA receptor modulators, an amide compound library established in the laboratory was used. High‐throughput screening was performed, and *K*_d_ values were calculated as described above. The compound with the most favorable *K*_d_ value was selected as a hit.

### Analysis of Binding Affinity Between Compounds and PYR1

The ITC and BLI were used to measure the binding affinities of the compounds to PYR1. BLI assays were performed using a ForteBIO Octet RED96 system equipped with specialized data acquisition and analysis software (Pall ForteBIO Corp., CA, USA). After loading PYR1 or mutant PYR1 onto Super Streptavidin (SSA) Dip and Read Biosensors, the biosensors were transferred to wells containing various concentrations of AM1, AMF4, pyrabactin, and diopyridin. The binding process included a baseline, association, and dissociation. Binding affinities were derived using data software (version 8.0, ForteBio). In addition, the binding affinities of ABA, compounds 26a, 145a, 260a, 361a, 368a, and 470a to PYR1 were determined using ITC. The titrimetric examination of PYR1 with these chemicals was executed using a meticulously equipped Micro Cal ITC200 microcalorimeter operating at 25 °C. A volume of 40 µL of these chemicals was meticulously titrated into a precisely matched buffer solution of PYR1 under constant stirring conditions of 1000 rpm, subsequent to which the equilibrium of the system was allowed to stabilize at 30 °C. Binding affinities were also calculated.^[^ ^29^ ^]^

### Fluorescent Competition on *Arabidopsis*

*Arabidopsis* seeds were surface sterilized using 70% (v/v) ethanol for 5 min and 2.6% (v/v) NaClO for 10 min, and washed seven times with deionized water. Seeds were subsequently sown onto 1/2 solid Murashige and Skoog (MS) plates, maintained in darkness at 4 °C for 3 days, and transferred to an incubator under a 16 h light/8 h dark cycle (21 °C, 50% humidity) for an additional 7 days period. Upon growth of two leaves, seedlings were immersed in lebactin solutions (100 µm) containing 150 mm NaCl, and with or without ABA, pyrabactin, AM1, and AMF4, then incubated for 72 h. Subsequently, the roots were photographed under a confocal microscope.

### Molecular Dynamics

To confirm binding confirmation between lebactin and PYR1, molecular dynamics analysis was performed using AMBER22. The crystal structure of PYR1 (PDB ID: 3K3K), a protein isolated from *Arabidopsis*, was retrieved from the RCSB Protein Databank. The protein system was immersed in a rectangular box with the TIP3P water model at a distance of 10 Å. The AMBER ff14SB and Gaff force fields were used for protein residues and ligands, respectively. The systems were energy minimized using the Sander module of AMBER22 to eliminate inappropriate interactions. Then, the 100 ns MD simulations were conducted in the PMEMD module. Each trajectory was replicated 10 times under different initial conditions. Binding modes were determined using the Cpptraj module. Snapshots from the stable trajectory of each system were used to calculate the MM/PBSA.

### Phenotypic Assays

Water losses of detached kidney‐bean leaves treated with 20 µm ABA and 100 µm diopyridin were quantified by monitoring fresh weight loss at designated time intervals, including 0, 0.5, 1, 1.5, 2, 2.5, 3, 3.5, and 4 h. The experiment was conducted at room temperature under 35% relative humidity. Water losses was quantified as the proportion of fresh weight at each distinct time interval relative to the initial fresh weight.

The methodology reported by Shang et al. (2016) was used to accurately measure stomatal apertures.^[^ ^30^ ^]^ Leaves from 7‐day‐old kidney beans were immersed into stomatal aperture solution (comprising 50 mm KCl, 10 µm CaCl_2_, 10 mm MES, pH 6.15), exposed to a light intensity of 150 µmol m^−2^s^−1^ in an incubator at 22 °C to induce full stomatal opening. ABA at 20 µm and diopyridin at 100 µm were added into the stomatal aperture solution for 1, 2, and 3 h. Subsequently, stomata were photographed under a microscope and stomatal width and length of stomata were measured. Stomatal aperture was determined by measuring the width‐to‐length ratio.

Seven‐day old kidney beans were subjected to withholding of water and sprayed with 40 µm ABA, 100 µm diopyridin, 0.05% DMSO, and 0.1% T‐80 water at 3‐day intervals. All plots were allocated an identical volume of soil, and the alignment of the plates and pots was altered daily to mitigate positional influences. Kidney beans were photographed 3, 6, 9, and 12 days after withholding of water, and 25 days after re‐watering. Meanwhile, the survival rate was calculated 25 days after re‐watering. Additionally, kidney beans treated with 20 µm ABA, and 100 µm diopyridin were imaged using an IR imager, and the leaf surface temperature was recorded.

### Phosphatase Activity Assay

A specific Ser/Thr phosphatase analysis apparatus (Promega) was used to precisely measure phosphatase activity, following a meticulously outlined protocol for the purpose. The reaction was conducted in a 50 µL reaction volume with PYR‐HAB1 at a ratio of 1:1 (0.1 µm:0.1 µm), and 0, 1.5625, 3.125, 6.25, 12.5, 25, and 50 µm of ABA and diopyridin were added into the reaction system.

### Mechanism of Turn on in Lebactin After Binding with PYR1

The mechanism underlying the brightness of lebactin after binding to PYR1 was elucidated using quantum mechanics/molecular mechanics (QM/MM). QM calculations (B3LYP/6‐31g^**^) were used to process free lebactin using Gaussian16. QM/MM calculations (Amber force field & B3LYP/6‐31g^**^) were used to process the PYR1‐lebactin binding using Gaussian 16 and Amber 22. Simultaneously, all calculations accounted for the effect of solvent water. The frontier molecular orbitals and corresponding energies of the lebactin‐free and lebactin‐PYR1 complexes were calculated.

### Statistical Analysis

All analyses were performed using SAS software (version 9.2, SAS Institute, Cary, NC, USA). All experiments were performed with at least three biological replicates. Data were shown as mean ± SE. Statistical significance was determined using Tukey's test, with value of ^*^ *p* < 0.05, ^**^ *p* < 0.01, and ^***^ *p* < 0.001.

## Conflict of Interest

The authors declare no conflict of interest.

## Supporting information



Supporting Information

Supporting Information

## Data Availability

The data that support the findings of this study are available from the corresponding author upon reasonable request.
