# Supplementary material for: High‐Throughput Fluorescence Screening Enables Globally Consistent Identification of ABA Signaling Modulators
Source: Adv Sci (Weinh). 2025 May 8;12(22):2417212. doi: 10.1002/advs.202417212 (PMC12165062; doi:10.1002/advs.202417212)
Supplement: Supplementary file 1 — Supporting Information [file ADVS-12-2417212-s002.docx]

**Supplementary Materials**

**High-Throughput Fluorescence Screening Enables Globally Consistent Identification of ABA Signaling Modulators**

Yang-Yang Gao,^a^ Chang-Xin Yang,^a^ Hong Wu,^a^ Jian-Hong Li,^a^ Ting Wen,^a^ Wei Wang,^a^ Zhi-Zheng Wang^c^, Hui-Min Chen,^b^ Rong-Jie Pei,^b^ Zhi-You Huang,^b^ Yu-Guo Zheng,^a,d^ Guang-Fu Yang,^b^ Xiang-Yang Li^a^*, Ge-Fei Hao^a, b^*

^a^*State Key Laboratory of Green Pesticide, Key Laboratory of Green Pesticide and Agricultural Bioengineering, Ministry of Education, Center for Research and Development of Fine Chemicals, Guizhou University, Guiyang 550025, P. R. China*

^b^*State Key Laboratory of Green Pesticide, Central China Normal University, Wuhan, 430079, P. R. China*

^c^*State Key Laboratory of Biocatalysis and Enzyme Engineering, School of Life Sciences, Hubei University, Wuhan, Hubei, PR China*

^d^*Key Laboratory of Chemical Synthesis and Environmental Pollution Control Remediation Technology, Minzu Normal University of Xingyi, Xingyi, Guizhou 562400, PR China*

*To whom correspondence should be addressed. [xyli1@gzu.edu.cn;](mailto:xyli1@gzu.edu.cn;) [gefei_hao@foxmail.com](mailto:gefei_hao@foxmail.com)

**Table S1** The fluorescence characteristics of 17 tested chemosensors

| **Chemosensors** | **Structures** | **Chemosensors (1 μM)** | | | **Chemosensors and PYR1** | | | **After incubation for 3 h** | |
| --- | --- | --- | --- | --- | --- | --- | --- | --- | --- |
|  |  | **Excitation (nm)** | **Emission (nm)** | **Fluorescence intensity** | **Emission (nm)** | **Fluorescence intensity** | **Increased times** | **Fluorescence intensity** | **Increased times** |
| **Compound 1a** |  | 330 | 565 | 19.81 | 488 | 280.81 | 14.18 | 280.32 | 14.18 |
| **Compound 2a** |  | 340 | 550 | 61.2 | 523 | 300.49 | 4.91 | - | - |
| **Compound 3a** |  | 335 | 540 | 30.24 | 515 | 237.38 | 7.85 | - | - |
| **Compound 4a** |  | 310 | 527 | 101.4 | - | - | - | - | - |
| **Compound 5a** |  | 325 | 565 | 43.82 | 522 | 578.71 | 13.21 | 312.24 | 7.13 |
| **Compound 6a** |  | 310 | 568 | 15.03 | 510 | 76.50 | 5.09 | - | - |
| **Compound 7a** |  | 370 | 530 | 20.56 | 530 | 97.04 | 4.72 | - | - |
| **Compound 8a** |  | 310 | 530 | 10.35 | 522 | 45.62 | 4.41 | - | - |
| **Compound 9a** |  | 310 | 525 | 55.28 | 531 | 123.88 | 2.24 | - | - |
| **Compound 10a** |  | 370 | 518 | 10.01 | 527 | 314.09 | 31.38 | - | - |
| **Compound 11a** |  | 310 | 557 | 20.99 | 515 | 108.10 | 5.15 | - | - |
| **Compound 12a** |  | 340 | 517 | 5.00 | 521 | 7.34 | 1.47 | - | - |
| **Compound 1b** |  | 310 | 540 | 10.20 | 472 | 90.98 | 8.92 | - | - |
| **Compound 2b** |  | 350 | 534 | 5.00 | 472 | 68.35 | 13.67 | - | - |
| **Compound 3b** |  | 330 | 529 | 42.43 | 519 | 68.76 | 1.62 | - | - |
| **Compound 4b** |  | 310 | 532 | 223.57 | - | - | - | - | - |
| **Compound 5b** |  | 350 | 532 | 112.24 | - | - | - | - | - |

**Table S2** The potential ABA receptor modulators obtained by high-throughput screening a several hundreds-amide compound library

| Compounds | F(GhitFluors)/*K*_d_/μM | ITC/*K*_d_/μM | BLI/*K*_d_/μM |
| --- | --- | --- | --- |
| 145a | 37.75 ± 1.31 | 170.94 ± 3.76 |  |
| 260a | 3.96 ± 0.4 | 7.59 ± 1.62 |  |
| 361a | 8.25 ± 0.25 | 7.06 ± 1.92 |  |
| 368a | 4.73 ± 0.37 | 14.89 ± 3.29 |  |
| 470a | 8.43 ± 0.75 | 9.51 ± 2.19 |  |
| 173b | 1.55 ± 0.11 |  | 9.50 ± 0.75 |

**Table S3** The advantages of diopyridin compared with known ABA agonists

| Compounds | Structural formula | Seed germination | Root growth |
| --- | --- | --- | --- |
| Pyrabactin |  | + | + |
| AM1 |  | + | + |
| AMF4 |  | + | + |
| Cyanabactin |  | + | + |
| Opabactin |  | + | + |
| Diopyridin |  | **-** | **-** |

Note: + Effect - No effect

**Scheme 1.** The synthesis route of lebactin

**Scheme 2.** The synthesis route of sulfonamide compounds without nitro.

**Scheme 3.** The synthesis route of sulfonamide compounds with nitro.

**Scheme 4.** The synthesis route of amide compounds

**5-(dimethylamino)-N-(pyridin-2-ylmethyl)naphthalene-1-sulfonamide** (**Lebactin**): ^1^H NMR (500 MHz, DMSO-*d_6_*) δ 8.58 (t, J = 6.3 Hz, 1H), 8.36 (d, J = 8.5 Hz, 1H), 8.29 – 8.24 (m, 2H), 8.05 (dd, J = 7.3, 1.1 Hz, 1H), 7.57 – 7.48 (m, 3H), 7.18 (dd, J = 14.8, 7.6 Hz, 2H), 7.10 – 7.06 (m, 1H), 4.08 (d, J = 6.3 Hz, 2H), 2.77 (s, 6H). ^13^C NMR (126 MHz, DMSO-*d_6_*) δ 157.63, 151.82, 149.01, 136.83, 136.52, 129.92, 129.52, 129.49, 128.88, 128.34, 124.01, 122.71, 121.89, 119.70, 115.59, 48.32, 45.60.

***N*-(4-bromo-2,6-dinitrophenyl)-4-(tert-butyl)benzenesulfonamide** (**145a**): Yellow solid; yield 39 %; mp 140.6-141.5 °C; ^1^H NMR (600 MHz, DMSO-*d_6_*) δ 10.92 (s, 1H), 8.52 (s, 2H), 7.55 (d, J = 8.4 Hz, 2H), 7.49 (d, J = 8.4 Hz, 2H), 1.30 (s, 9H). ^13^C NMR (100 MHz, DMSO) δ 156.26, 148.65, 136.09, 131.47, 126.08, 125.99, 121.65, 120.55, 34.93, 30.76. HRMS (ESI): m/z [M+Na]+ calcd for, C_16_H_16_BrN_3_O_6_S : 479.9841 ; found: 479.9829.

***N*-(4-bromo-2,6-dinitrophenyl)-3-fluorobenzenesulfonamide (260a):** Yellow solid; yield 35 %; mp 119.4-120.3 °C; ^1^H NMR (600 MHz, DMSO-*d_6_*) *δ* 8.52 (s, 2H), 7.58 (m, 2H), 7.43 (d, *J* = 7.8 Hz, 1H), 7.38 (d, *J* = 9.0 Hz, 1H). ^13^C NMR (100 MHz, DMSO) *δ* 171.66, 148.47, 141.54, 141.48, 131.67, 131.56, 122.51, 121.47, 120.69, 120.39, 120.17. HRMS (ESI): m/z [M+Na]+ calcd for, C12H7BrFN3O6S : 441.9121 ; found: 441.9143.

**N-(4-bromo-2,6-dinitrophenyl)-3-chlorobenzenesulfonamide (361a)**: Yellow solid; yield 37 %; mp 155.6-156.8 °C; 1H NMR (400 MHz, DMSO-*d_6_*) δ 8.50 (s, 2H), 7.74 (d, *J* = 5.4 Hz, 1H), 7.62 – 7.46 (m, 3H). 13C NMR (150 MHz, DMSO) δ 148.75, 141.73, 133.99, 133.28, 131.83, 131.51, 125.97, 125.18, 121.73, 120.90. HRMS (ESI): m/z [M+Na]^+^ calcd for, C_12_H_7_BrClN_3_O_6_S : 457.8825 ; found: 457.8851.

***N*-(4-bromo-2,6-dinitrophenyl)-2-methylbenzenesulfonamide (368a):** Yellow solid; yield 32 %; mp 161.2-162.5 °C; ^1^H NMR (600 MHz, DMSO-*d_6_*) δ 8.52 (s, 2H), 7.58 (m, 2H), 7.43 (d, *J* = 7.7 Hz, 1H), 7.38 (d, *J* = 8.7 Hz, 1H), 1.91 (s, 3H). ^13^C NMR (150 MHz, DMSO) δ 149.71, 137.60, 137.19, 133.51, 132.85, 131.60, 128.27, 126.44, 121.73, 121.39, 20.04. HRMS (ESI): m/z [M+Na]+ calcd for, C13H10BrN3O6S: 437.9371 ; found: 437.9357.

***N*-(4-bromo-2,6-dinitrophenyl)-2-chlorobenzenesulfonamide (470a):** Yellow solid; yield 31 %; mp 184.2-185.1 °C; ^1^H NMR (600 MHz, DMSO-*d_6_*) *δ* 8.50 (s, 2H), 7.77 (d, *J* = 7.8 Hz, 1H), 7.64 (s, 2H), 7.46 (d, *J* = 4.8 Hz, 1H). ^13^C NMR (150 MHz, DMSO) δ 149.06, 137.70, 134.74, 131.92, 131.48, 131.21, 130.10, 127.74, 122.15, 120.88. HRMS (ESI): m/z [M+Na]+ calcd for, C12H7BrClN3O6S : 457.8825 ; found: 457.8842.

***N*-(2(2,6-dioxopiperidin-3-yl)-1,3-dioxoisoindolin-4-yl)-3,4-dimethoxybenzamide (173b):** Yellow solid; yield 36%; mp 172.3-173.5 °C; ^1^H NMR (400 MHz, DMSO-*d_6_*) δ 11.17 (s, 1H), 10.32 (s, 1H), 8.60 (d, *J* = 8.3 Hz, 1H), 7.94-7.87 (m, 1H), 7.65 (d, *J* = 7.2Hz, 1H), 7.58 (dd, *J* = 8.4, 2.1 Hz, 1 H), 7.53 (d, *J* = 2.0 Hz, 1 H), 7.21-7.16 (m, 1H), 5.19 (dd, *J* = 12.9, 5.4 Hz, 1 H), 3.86 (s, 6H), 2.91 (ddd, J = 17.6, 14.1, 5.4 Hz, 1H), 2.63 (d, J = 16.7 Hz, 2H), 2.21-2.01 (m, 1H). ^13^C NMR (125 MHz, DMSO-d6) δ: 173.20, 170.19, 168.77, 167.16, 164.97, 152.98, 149.27, 137.39, 136.78, 131.82, 126.41, 125.96, 120.99, 118.92, 118.02, 111.91, 111.15, 56.20, 49.46, 31.41, 22.48. C22H19N3O7; found: 460.11.

**
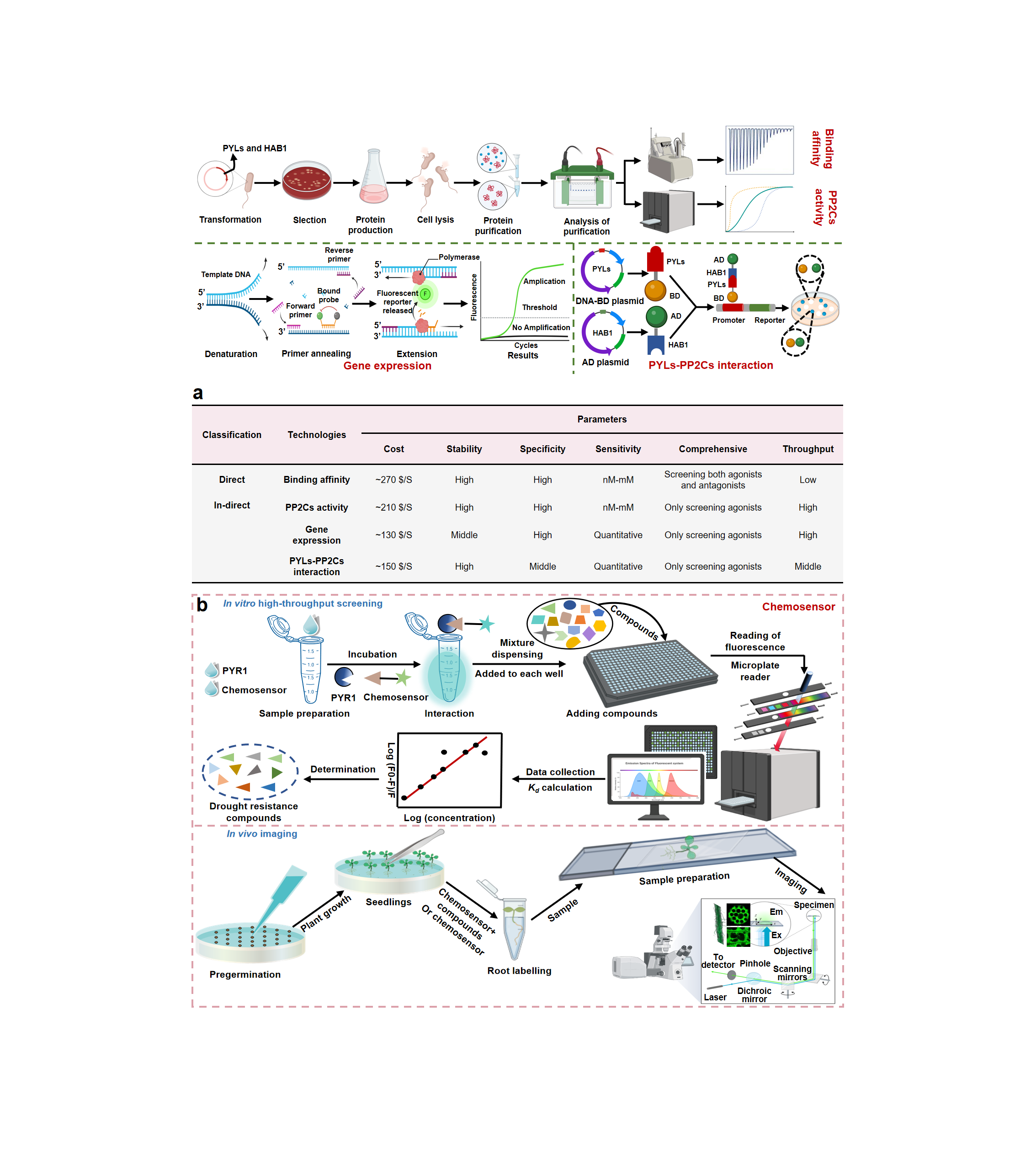
**

**Figure S1** The workflow of traditional techniques including measuring binding affinity between PYLs and compounds, PP2Cs activity, ABA-responsive gene expression, and PYLs-PP2Cs interaction for the discovery of ABA receptor modulators.


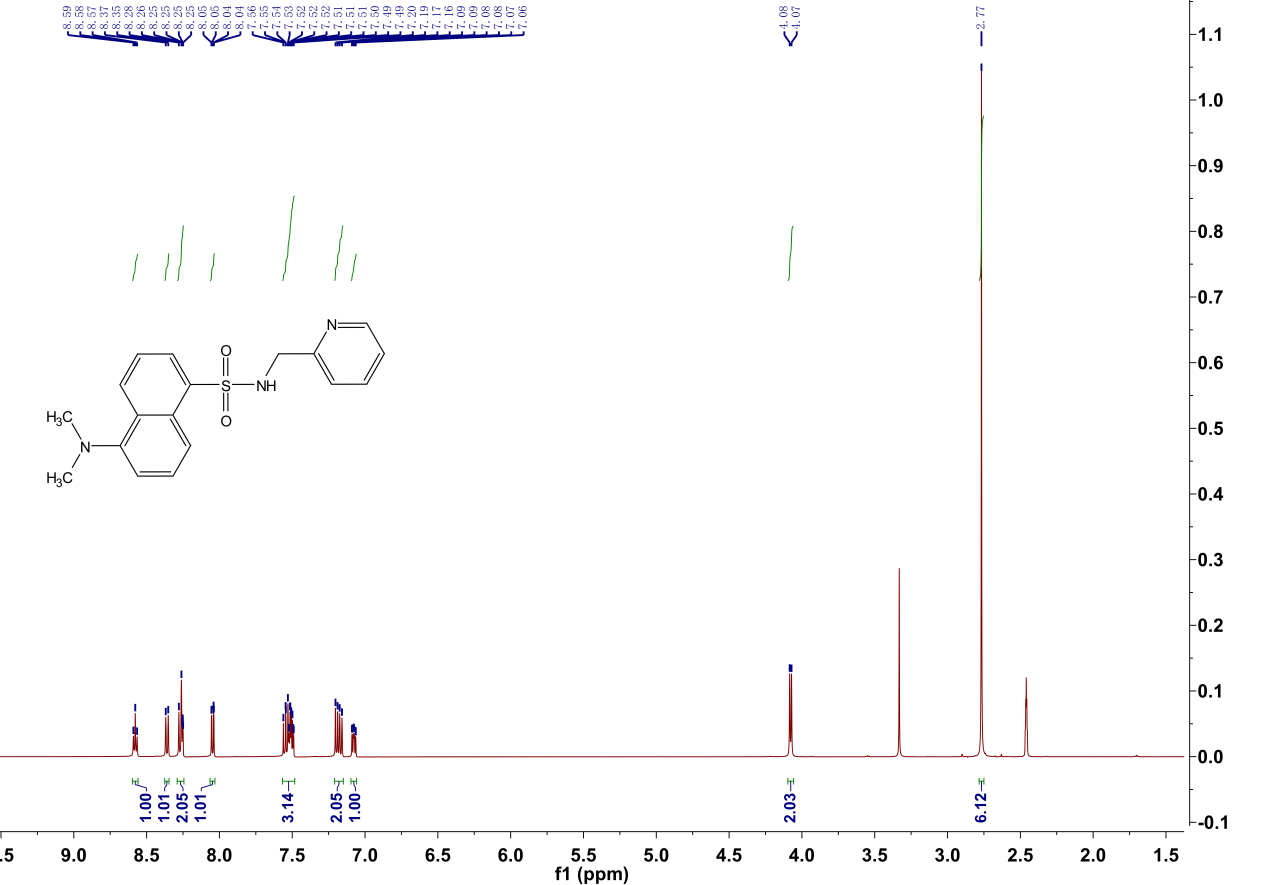


**Figure S2** ^1^H NMR spectrum of lebactin in DMSO-d6


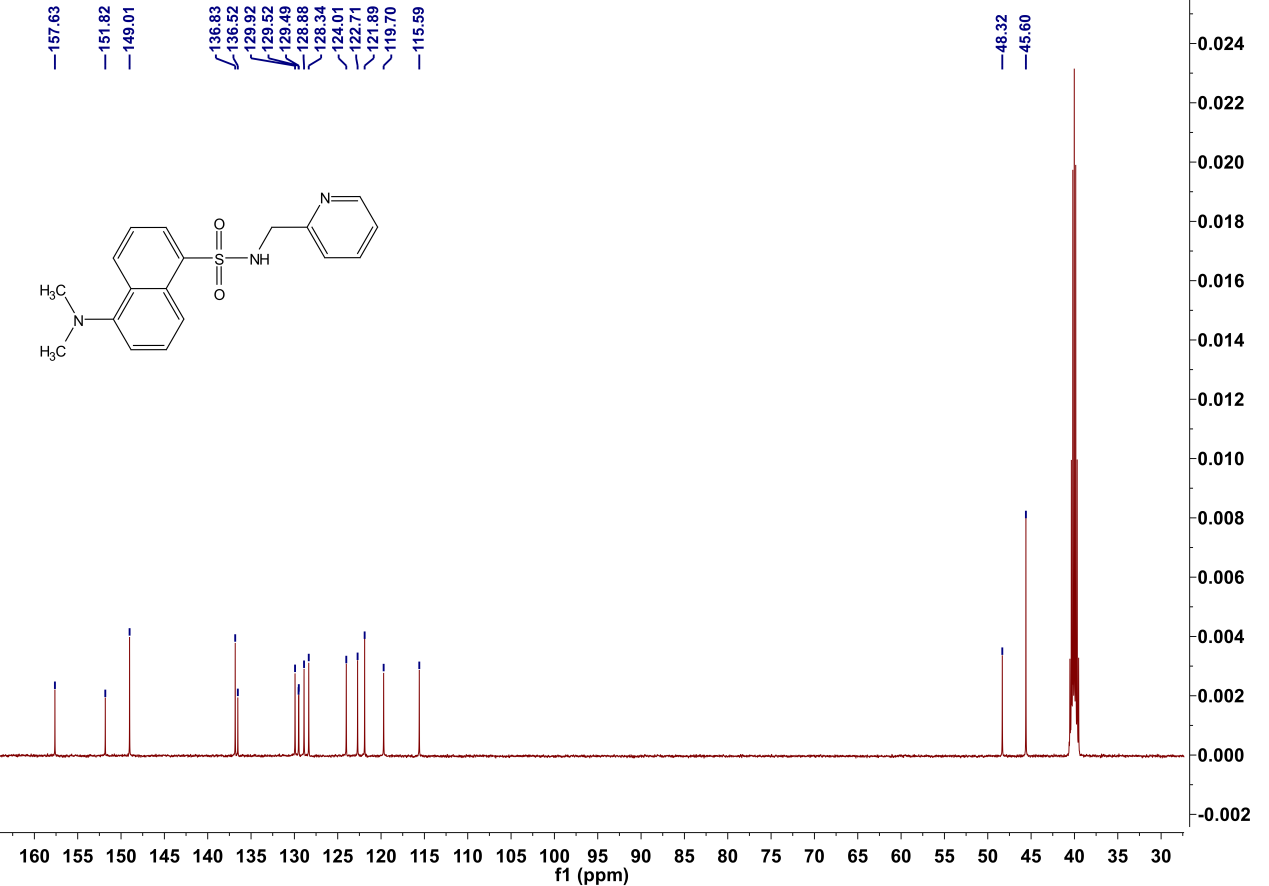


**Figure S3** ^13^C NMR spectrum of lebactin in DMSO-d6

**
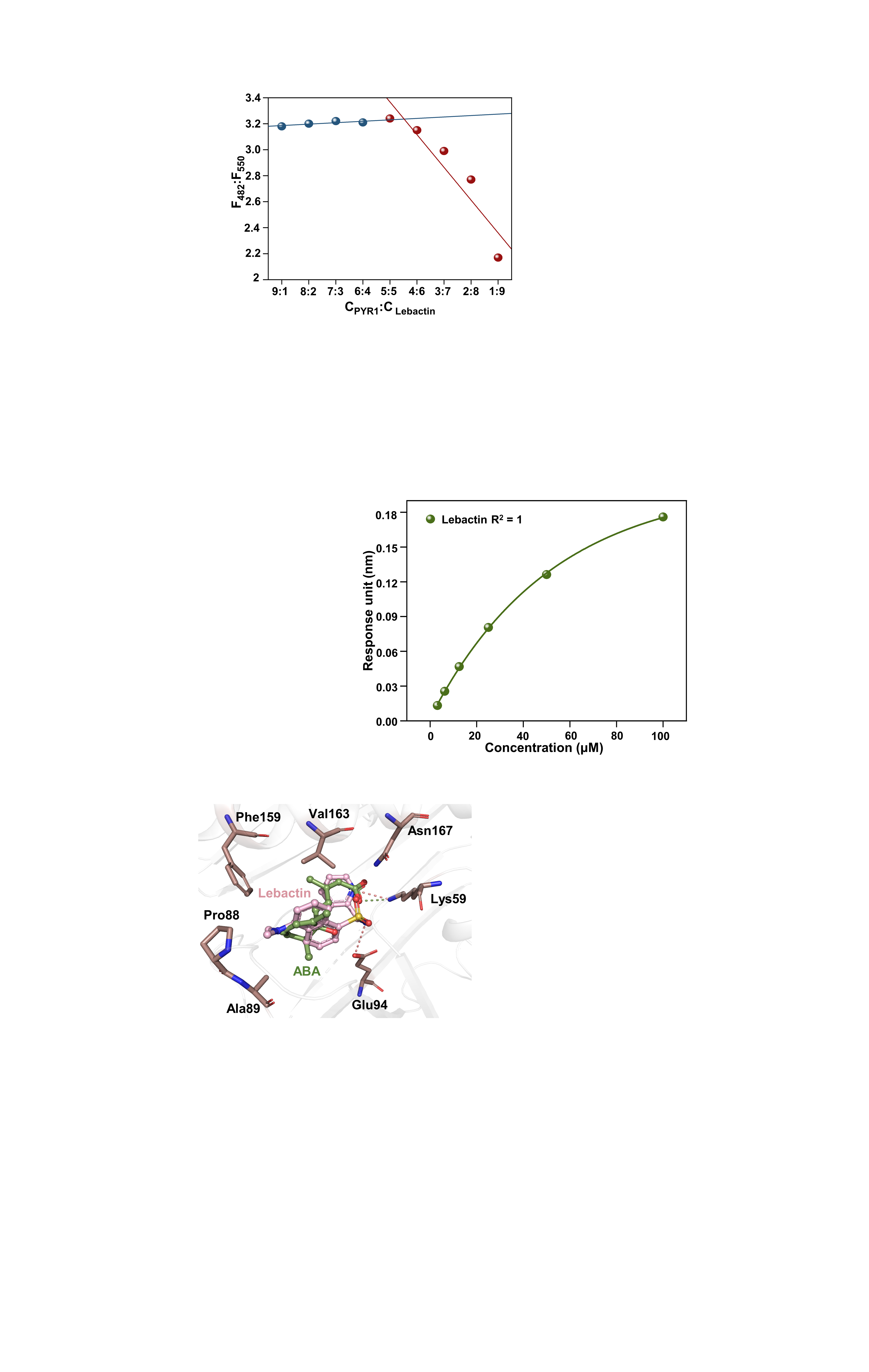
**

**Figure S4** The Job’s plot curve of lebactin-PYR1 at different ratios of 10:0, 9:1, 8:2, 7:3, 6:4, 5:5, 4:6, 3:7, 2:8, 1:9 and 0:10. The intersection point after linear fitting data synthesis showed that the optimal binding ratio of lebactin-PYR1 is 1:1


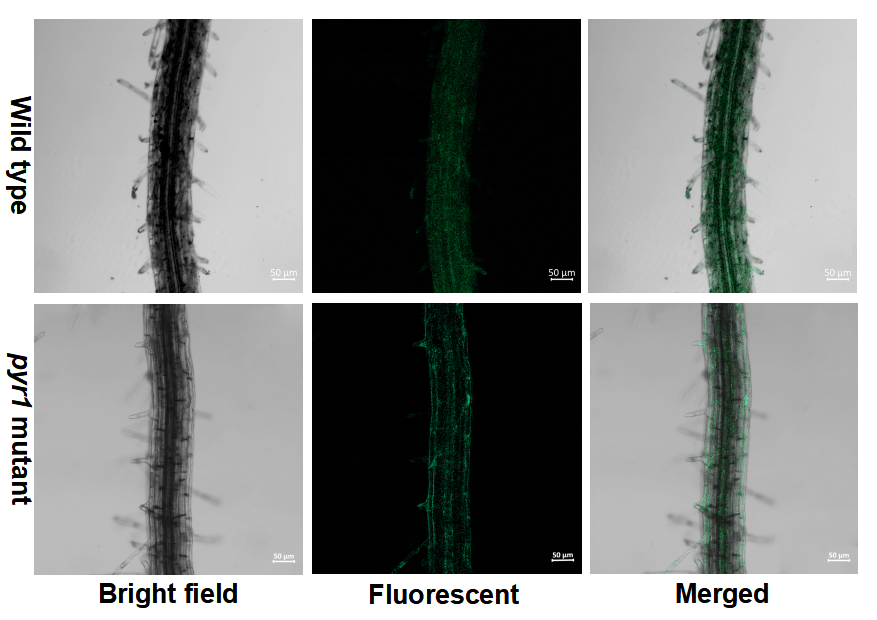


**Figure S5** Confocal imaging of 7-day old *Arabidopsis* roots for *pyr1* mutant treated with lebactin


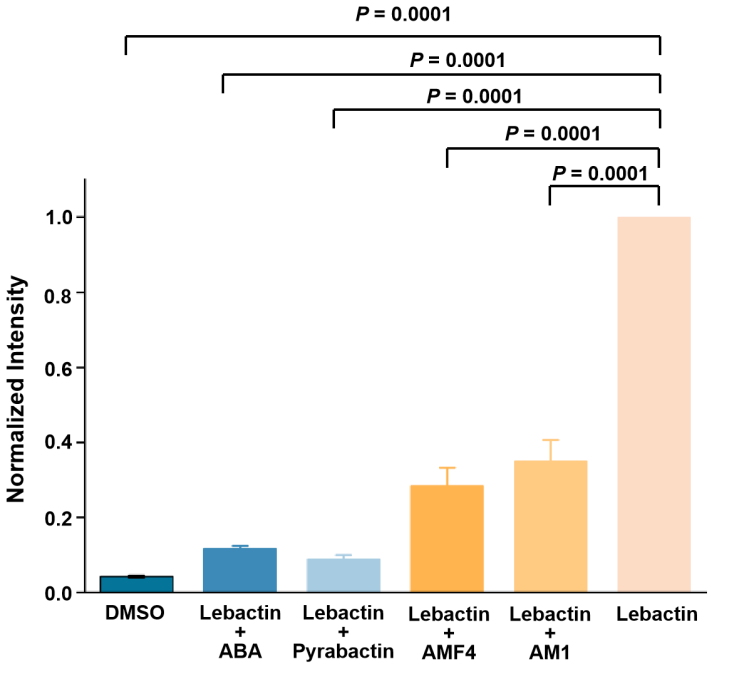


**Figure S6** The normalized intensity of root imaging. All treatments significantly decreased the fluorescence intensity compared with the lebactin treated group. *n* = 3 for each group; the error bars were SE.


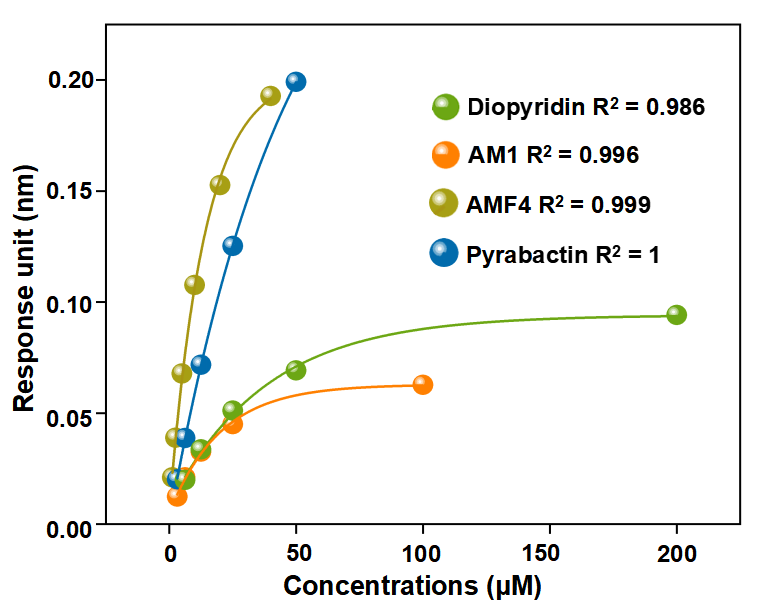


**Figure S7** The relationship between the concentrations of AM1, AMF4, pyrabactin, diopyridin and their response unit in BLI assays

**Figure S8** ^1^H NMR spectrum of 145a in DMSO-d6

**Figure S9** ^13^C NMR spectrum of 145a in DMSO-d6

**Figure S10** ^1^H NMR spectrum of 260a in DMSO-d6

**Figure S11** ^13^C NMR spectrum of 260a in DMSO-d6

**Figure S12** ^1^H NMR spectrum of 361a in DMSO-d6

**Figure S13** ^13^C NMR spectrum of 361a in DMSO-d6

**Figure S14** ^1^H NMR spectrum of 368a in DMSO-d6

**Figure S15** ^13^C NMR spectrum of 368a in DMSO-d6

**Figure S16** ^1^H NMR spectrum of 470a in DMSO-d6

**Figure S17** ^13^C NMR spectrum of 470a in DMSO-d6


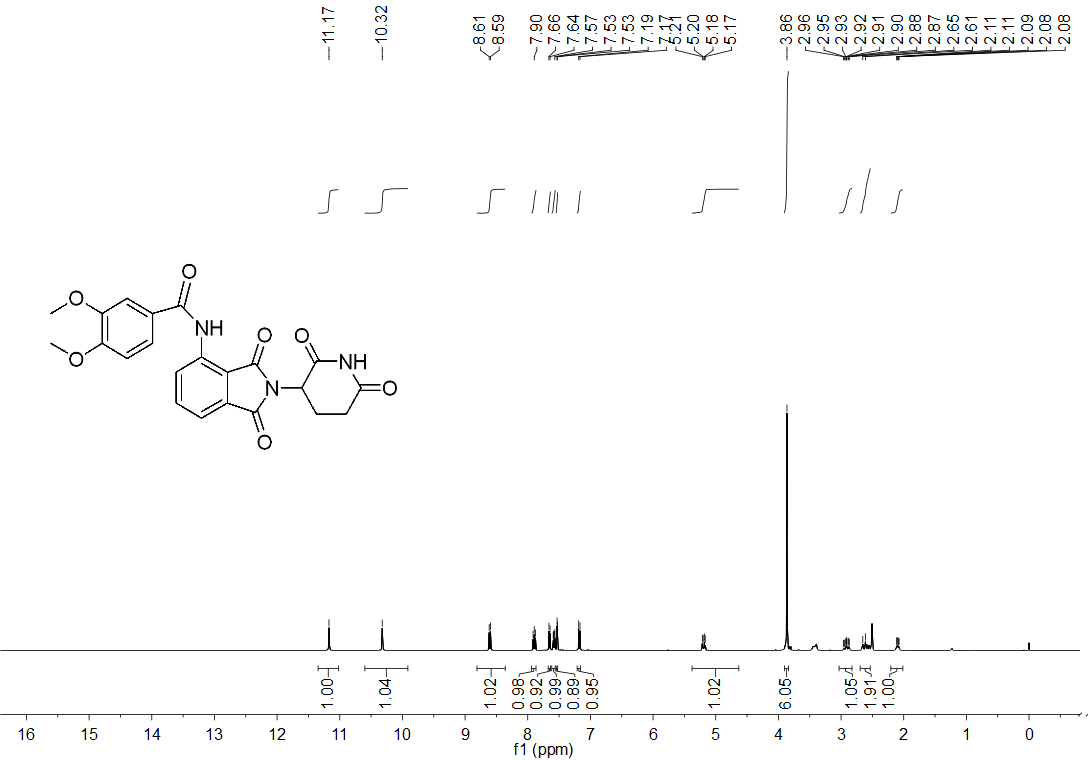


**Figure S18** ^1^H NMR spectrum of 173b in DMSO-d6


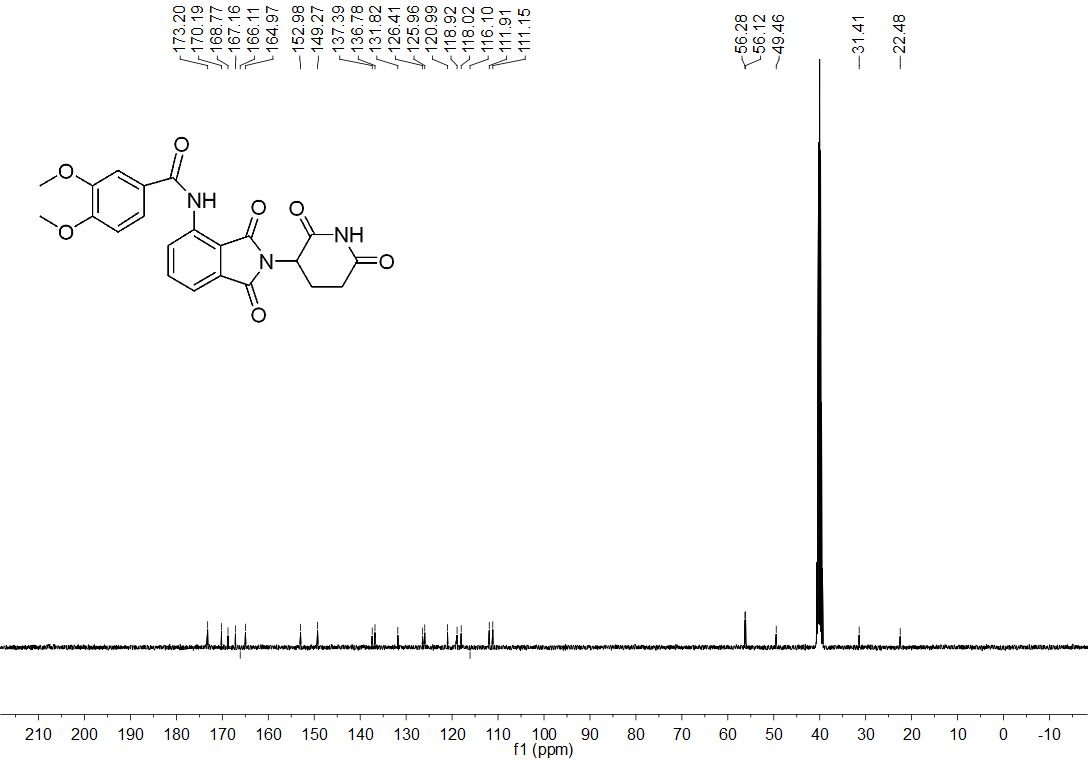


**Figure S1****9** ^13^C NMR spectrum of 173b in DMSO-d6


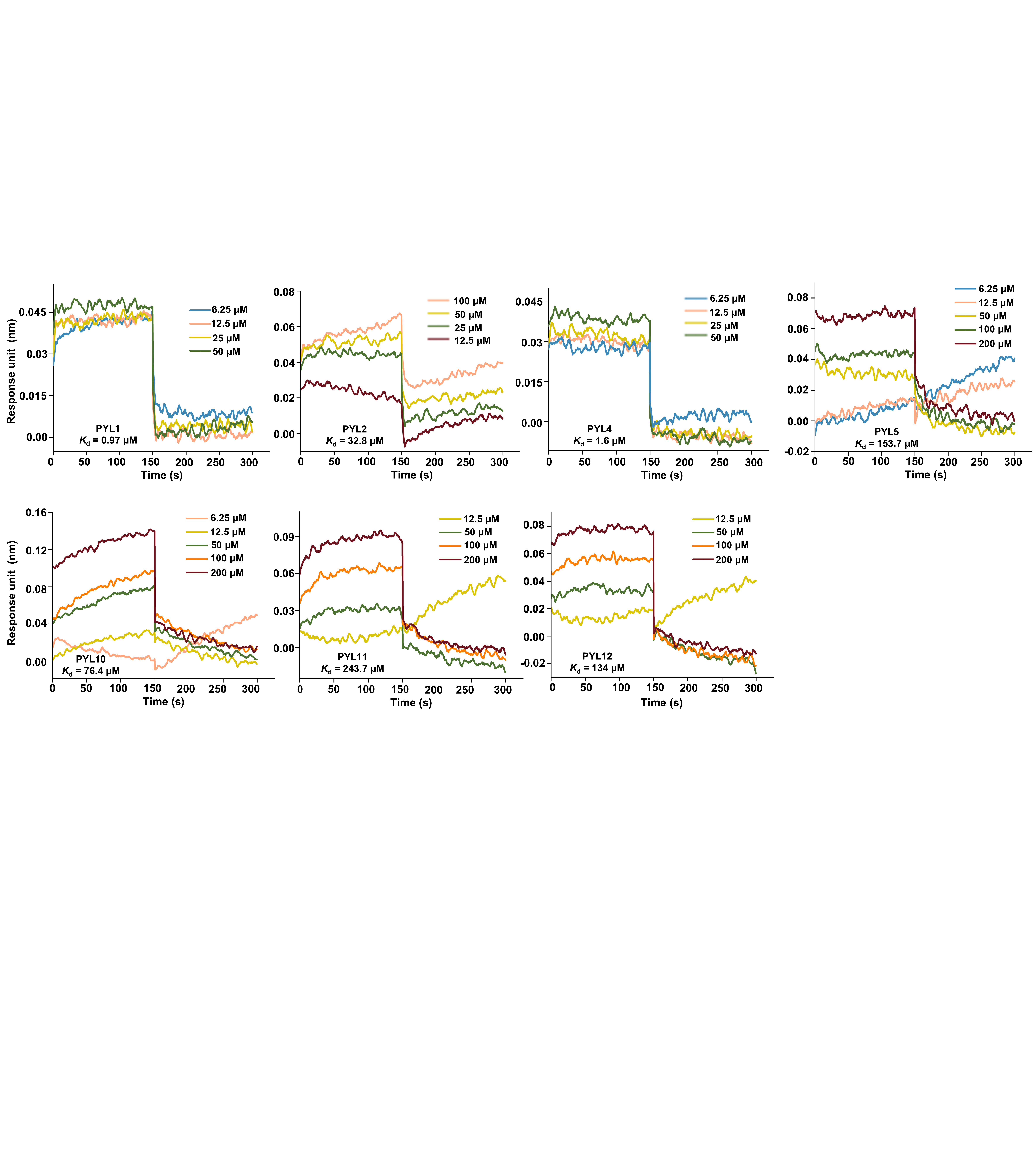


**Figure S20** The binding affinity of diopyridin with other representative PYLs


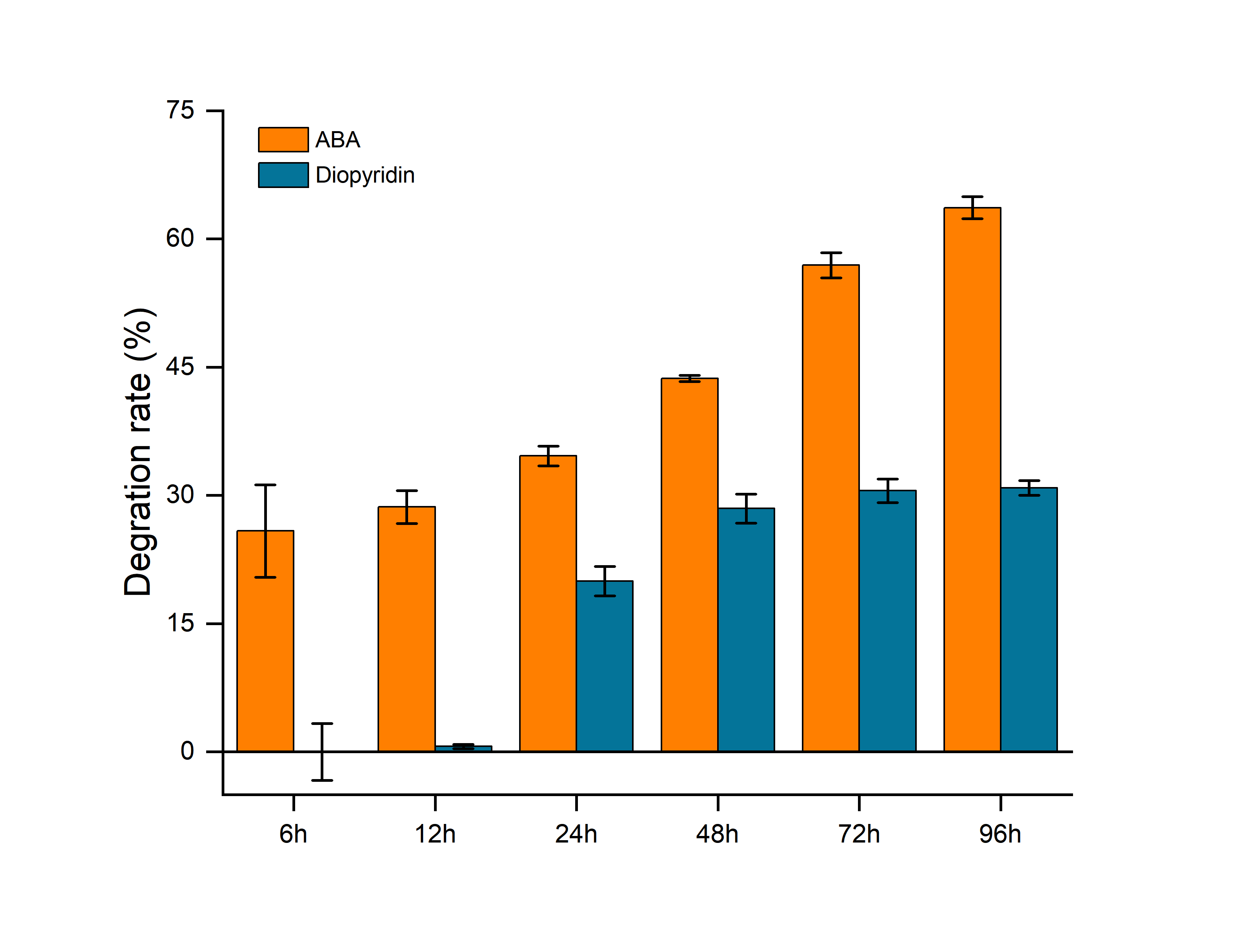


**Figure S21** The degradation rate of ABA and diopyridin under UV condition


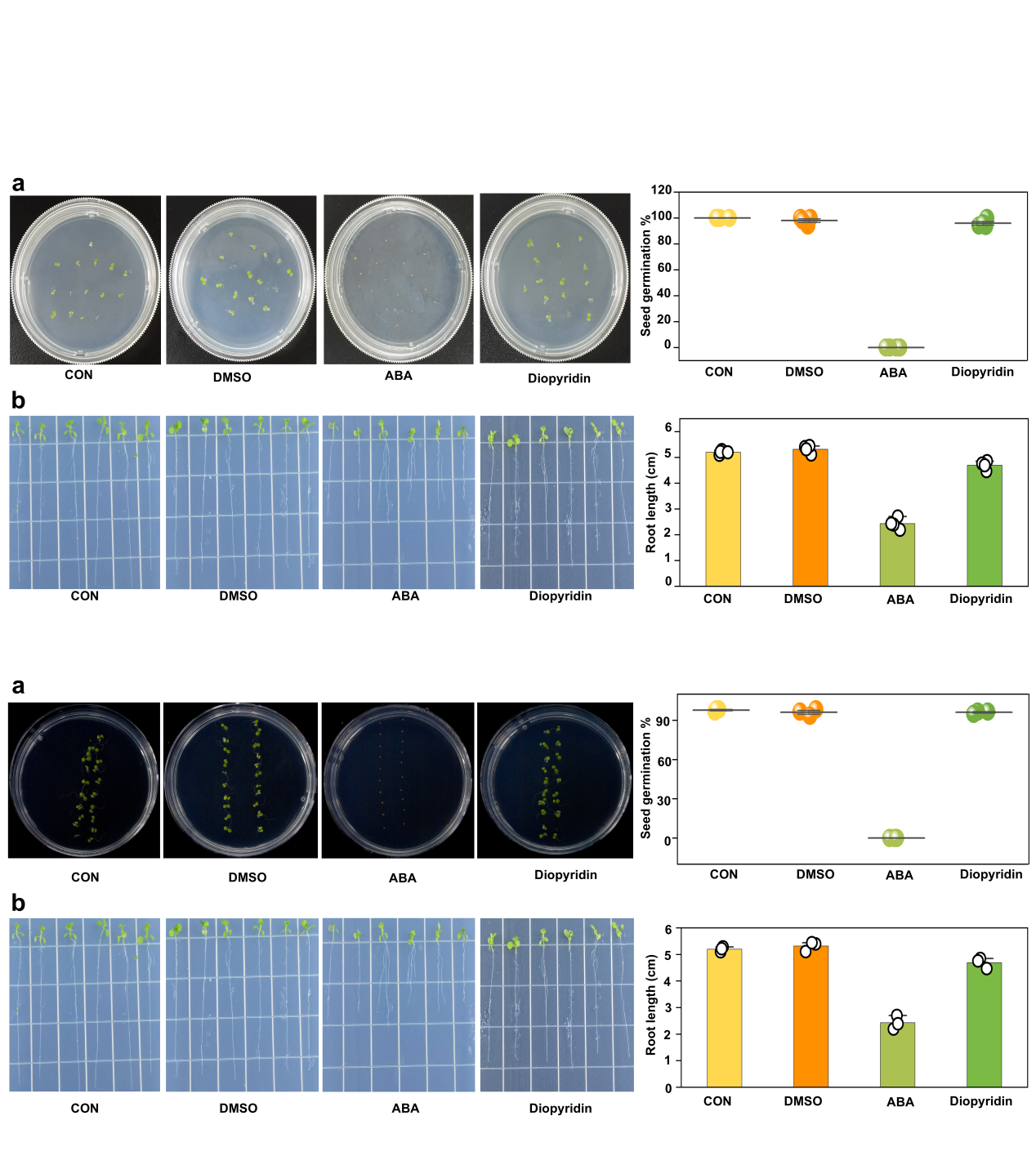


**Figure S22** The effect of diopyridin on the seed germination (a) and root growth (b)
